# The mediating roles of the oral microbiome in saliva and subgingival sites between e-cigarette smoking and gingival inflammation

**DOI:** 10.1186/s12866-023-02779-z

**Published:** 2023-02-02

**Authors:** Bongsoo Park, Hyunwook Koh, Michael Patatanian, Hermes Reyes-Caballero, Ni Zhao, Jill Meinert, Janet T. Holbrook, Leah I. Leinbach, Shyam Biswal

**Affiliations:** 1 Department of Environmental Health and Engineering, Johns Hopkins School of Public Health, 615 N Wolfe St, Baltimore, MD 21205 USA; 2 Epigenetics and Stem Cell Aging, Translational Gerontology Branch, National Institute On Aging, National Institute of Health, Baltimore, MD 21224 USA; 3 Department of Applied Mathematics and Statistics, The State University of New York, Korea Incheon, 21985 South Korea; 4 Department of Biostatistics, Johns Hopkins School of Public Health, Baltimore, MD 21205 USA; 5 Department of Epidemiology, Johns Hopkins School of Public Health, Baltimore, MD 21205 USA; 6 Department of Health Policy and Management, Johns Hopkins School of Public Health, Baltimore, MD 21205 USA; 7 Johns Hopkins School of Medicine, Baltimore, MD 21205 USA

**Keywords:** Electronic cigarette, Oral microbiome, Saliva microbiome, Subgingival microbiome, Gingival inflammation, Periodontal disease

## Abstract

**Background:**

Electronic cigarettes (ECs) have been widely used by young individuals in the U.S. while being considered less harmful than conventional tobacco cigarettes. However, ECs have increasingly been regarded as a health risk, producing detrimental chemicals that may cause, combined with poor oral hygiene, substantial inflammation in gingival and subgingival sites. In this paper, we first report that EC smoking significantly increases the odds of gingival inflammation. Then, through mediation analysis, we seek to identify and explain the mechanism that underlies the relationship between EC smoking and gingival inflammation via the oral microbiome.

**Methods:**

We collected saliva and subgingival samples from 75 EC users and 75 non-users between 18 and 34 years in age and profiled their microbial compositions via 16S rRNA amplicon sequencing. We conducted raw sequence data processing, denoising and taxonomic annotations using QIIME2 based on the expanded human oral microbiome database (eHOMD). We then created functional annotations (i.e., KEGG pathways) using PICRUSt2.

**Results:**

We found significant increases in α-diversity for EC users and disparities in β-diversity between EC users and non-users. We also found significant disparities between EC users and non-users in the relative abundance of 36 microbial taxa in the saliva site and 71 microbial taxa in the subgingival site. Finally, we found that 1 microbial taxon in the saliva site and 18 microbial taxa in the subgingival site significantly mediated the effects of EC smoking on gingival inflammation. The mediators on the genus level, for example, include *Actinomyces*, *Rothia, Neisseria,* and *Enterococcus* in the subgingival site. In addition, we report significant disparities between EC users and non-users in the relative abundance of 71 KEGG pathways in the subgingival site.

**Conclusions:**

These findings reveal that continued EC use can further increase microbial dysbiosis that may lead to periodontal disease. Our findings also suggest that continued surveillance for the effect of ECs on the oral microbiome and its transmission to oral diseases is needed.

**Supplementary Information:**

The online version contains supplementary material available at 10.1186/s12866-023-02779-z.

## Background

Smoking remains the most common cause of preventable death in the U.S. [1]. Because of this, cigarette smoking has been the target of one of the most robust public health campaigns in the U.S., ultimately leading to a decline in traditional cigarette use, particularly among younger members of the population [2]. Between 2011 and 2017, the proportion of high school students reporting cigarette use within the last thirty days declined from 15.8% to 7.6% [2]. However, at the same time, the use of non-combusted tobacco products, particularly electronic cigarettes (ECs), has increased.

ECs were initially introduced as tobacco cessation aids, but they have later found traction as a primary method of nicotine delivery, particularly among tobacco naïve adolescents and younger adults. Unlike traditional cigarettes, in which tobacco is burned releasing nicotine along with other combustion byproducts into the oral cavity and respiratory tracts of users, ECs deliver nicotine via an aerosolized vape liquid. EC aerosol contains nicotine at highly variable concentrations (approximately 1.2 mg/cartridge) [3], as well as other substances including acetaldehyde, acrolein, formaldehyde, propylene glycol, glycerin, ethanol, acetol, and propylene oxide [4, 5]. While the harms associated with traditional cigarette use are well-understood and predominantly secondary to exposure to combustion products, less is known about the local and systemic effects of aerosolized nicotine and other components of vape liquid, particularly over time. Despite these unknowns, EC use is now more common than cigarettes among adolescents in the U.S., with an increase in use from 1.5% of high school students in 2011 to 11.3% in 2016 [6]. The CDC reports a 14.4% increase in EC sales between 2014 and 2015 alone, with a concurrent 307.7% increase in the sale of vape liquids [7]. As of 2019, almost 21% of high school-age adolescents reported use of electronic cigarettes in the preceding month [8].

The increasing use of ECs, and subsequent exposure to nicotine, particularly among younger members of the population, is of concern for several reasons. First, ECs may act as a bridge to traditional combustion-based products [9]. ECs are marketed in a way that is appealing to younger people; vaporizers are designed to resemble devices other than cigarettes, including USBs and pens, bypassing some of the stigma that remains associated with traditional cigarettes. Second, exposure to the contents of vape liquid may be harmful in and of itself. Aerosolized components of vape liquid have been shown to adhere to the structures of the upper GI and respiratory tracts, including the oral cavity, nasal cavity and lungs, all sites known to have complex microbiomes [10–12].

How the exposure influences the microbial environments is of both scientific and clinical concern. Of particular interest in this paper are the effects of ECs on the oral microbiome composition in both the saliva and subgingival sites, and the mediating roles of the oral microbiome to gingival inflammation. The oral cavity is a main entryway through which many microbial species colonize the respiratory and gastrointestinal tracts [13]. Consisting of over 700 known species of bacteria, the oral cavity has the second largest microbial population after the gut microbiome, sharing characteristics with the microbiomes of the respiratory, gastrointestinal (GI), and genitourinary (GU) systems as well as characteristics unique to itself [14, 15]. The mouth with its various niches is an exceptionally complex habitat where microbes colonize the hard surfaces of the teeth and the soft tissues of the oral mucosa [15]. This complex system of viral, bacterial, and fungal species interacts to maintain a state of health as well as promote pathology. The microbial profile of the oral cavity is dynamic throughout the lifespan with shifts in populations consequent to diet, medication exposure, host immune function, oral hygiene practices, and exposure to cigarettes and other nicotine products [16]. Especially, the exposure to cigarettes and other nicotine products is thought to disrupt oral microbial communities in favor of the proliferation of pathogenic organisms leading to inflammation and disease [17].

Smoking tobacco use is known to favor the growth of pathogenic bacteria within the oral cavity through a shift from commensal aerobic bacteria to anaerobic species [18]. Such species have been associated not only with the development of oral diseases such as dental caries and periodontitis, but also systemic illnesses such as infective endocarditis, atherosclerosis, poor diabetes control, and rheumatoid arthritis [19]. Kim et. al recently published results of an in vitro study examining the effect of EC exposure on *Streptococcus mutans* (the organisms primarily responsible for dental caries), suggesting that EC use promotes biofilm deposition favorable to pathogenic bacterial growth [10]. Murine modeling of the effects of EC exposure on lung tissue suggests a decreased immune response to microbial stressors, including an impaired bactericidal capacity of neutrophils and a reduction in the cytokines involved in T cell-mediated immunity (IFN-gamma, TNF-alpha, IL17a) [20]. It appears reasonable to consider that a similar process may occur within the mucosal surfaces of the oral cavity. A recent study by Pushalkar et al*.* showed that the abundance of *Porphyromonas* and *Veillonella* genera was increased in the oral saliva samples of EC users, and epithelial cells exposed to EC aerosols appear to be more susceptible to microbial infection, providing a potential underlying immune compromising mechanism for higher inflammatory risk [21]. They also reported that salivary cytokines such as interleukin (IL)-6 and IL-1 β were elevated in EC users compared to non-users [21]. Another study published by Ganesan et al*.* also describes microbial dynamics, immunoinflammatory response, and expression change in subgingival plaque samples after EC exposure [22]. Prior research published by Thomas *et. al.* also indicates an association between EC use and poor oral health, suggesting a possible microbial shift (e.g. *Treponema*, *Saccharibacteria*, and *Porphyromonas*) favoring periodontal disease [23].

**Fig. 1:**
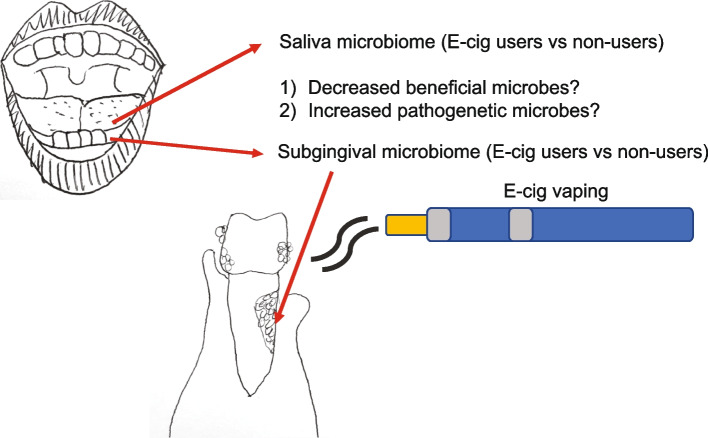
The illustration of saliva and subgingival sites, gingival inflammation, and EC vaping

This study aims to further elucidate any changes to the oral microbiome (both the salivary and subgingival environments) and, if present, whether these changes may lead to any clinical indications of poor oral health [Fig. 1]. For this, we first report that EC use significantly increases the odds of gingival inflammation (see *Study subjects and the effects of ECs on gingival inflammation*). Then, we performed mediation analyses to assess if EC use changes the oral microbiome (see *α-diversity analysis, β-diversity analysis, Taxonomic differential abundance analysis*) which in turn affects gingival inflammation (see *Mediation analysis*) when controlling for common risk factors. For our taxonomic analyses, we especially highlight our discoveries at the genus (not species) level, while our discoveries at the species level still tend to be notable references, because taxonomic annotations are precise from phylum to genus in the bacterial kingdom for the 16S rRNA amplicon sequencing [24].

Additionally, we performed differential abundance analyses for functional annotations using Kyoto Encyclopedia of Genes and Genomes (KEGG) pathways (see *Functional differential abundance analysis*) [25]. We finally discuss the implications of these findings and conclude that continued EC use by the young generation can further increase microbial dysbiosis, which may consequently cause periodontal disease and other pro-inflammatory conditions later in life.

## Results

### Study subjects and the effect of ECs on gingival inflammation

To assess the in vivo influence of ECs on the oral microbiome, we collected saliva and subgingival samples from 75 EC users and 75 non-users and profiled their microbial compositions via 16S rRNA amplicon sequencing. Table 1 shows the characteristics of the study subjects in terms of age, gender, the frequency of brushing teeth, and the clinical evidence of gingival inflammation. The EC users were slightly younger than the non-users in mean, first quartile, median and third quartile ages [Table 1]. The EC users were also more likely to be males: 81.33% (61 subjects) of the EC users are males, while 73.33% (55 subjects) of the non-users are males [Table 1]. The EC users brush teeth less frequently than the non-users [Table 1]. Thus, to control for potential confounding effects, we included age, gender, and the frequency of brushing teeth as covariates in the following data analyses.

**Table 1:** Descriptive table for the characteristics of study subjects

		EC users				Non-users			
		Mean	Median	Min, Max	IQR	Mean	Median	Min, Max	IQR
Age (unit: years)		23.84	23	18, 34	20, 27	24.40	25	18, 34	21, 28
		# subjects		% subjects		# subjects		% subjects	
Gingival Inflammation	Absent	30		40.00		47		62.67	
	Present	42		56.00		27		36.00	
Gender	Male	61		81.33		55		73.33	
	Female	14		18.66		20		26.67	
Freq. of brushing teeth	< 1	8		10.67		1		1.33	
	1	29		38.67		16		21.33	
	> 1	38		50.67		58		77.33	

^*^ Freq. of brushing teeth: How often do you brush your teeth? < 1: less than once per day, 1: once per day, > 1: Twice or more per day

**Fig. 2:**
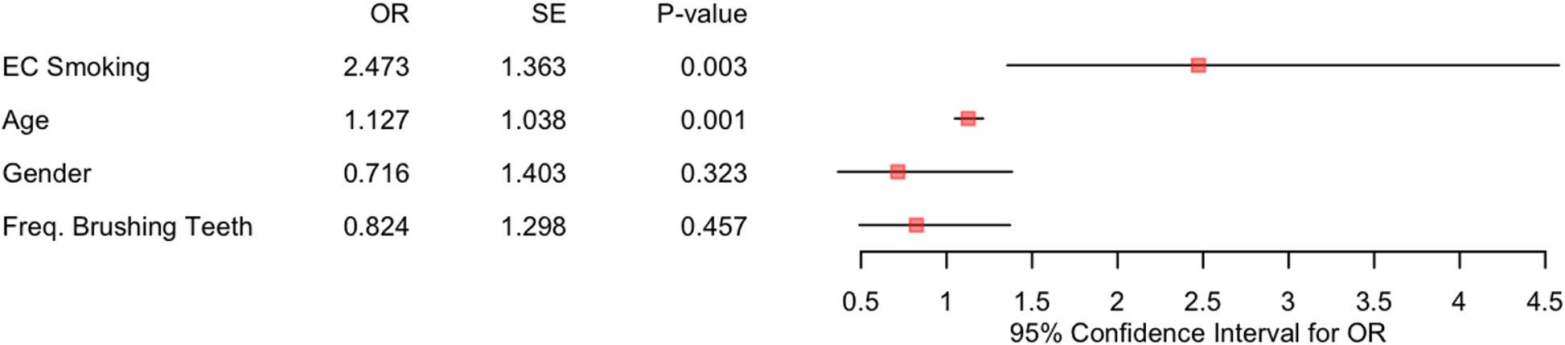
The effects of EC smoking and other covariates (age, gender, and the frequency of brushing teeth) on gingival inflammation. *EC Smoking represents our major finding on the effect of EC smoking on gingival inflammation while adjusting for age, gender, and the frequency of brushing teeth. Age, Gender, and Freq. Brushing Teeth represent the effects of age, gender, and the frequency of brushing teeth, respectively

We found that the EC users are more likely to have clinical evidence of gingival inflammation: 56% (42 subjects) versus 36% (27 subjects) of the non-users [Table 1]. We assessed the effect of EC smoking on gingival inflammation while adjusting for age, gender, and the frequency of brushing teeth [Fig. 2]. Our results indicate that EC smoking significantly increases the odds of gingival inflammation while adjusting for age, gender, and the frequency of brushing teeth (Odds ratio: 2.473 > 1, *P*-value: 0.003 < 0.05) [Fig. 2].

In the following sections, we describe the process underlying the association of EC smoking on gingival inflammation via the oral microbiome in saliva and subgingival sites, respectively. First, we assessed the disparity in microbial composition between EC users and non-users with respect to α-diversity, β-diversity, and taxonomic relative abundance while adjusting for age, gender, and the frequency of brushing teeth (see the sections, *α-diversity analysis*, *β-diversity analysis*, and *Taxonomic differential abundance analysis*). We then performed mediation analyses [26] to find microbial taxa that transmit the effect of EC smoking on gingival inflammation (see the section, *Mediation analysis*).

### α-diversity analysis

**Fig. 3:**
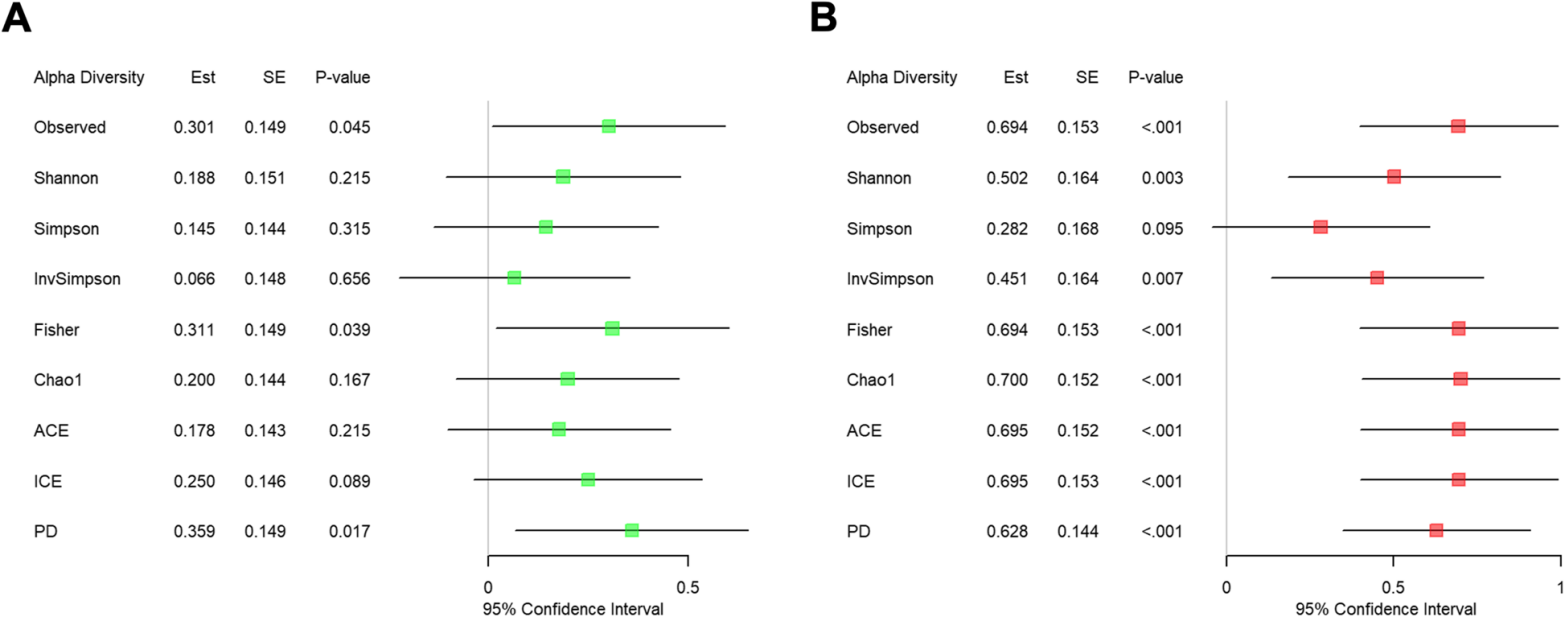
The results from α-diversity analysis. The fitted random effects model to assess the disparity in each α-diversity index between EC users and non-users while adjusting for age, gender and the frequency of brushing teeth in the saliva (**A**) and subgingival (**B**) sites, respectively. *Est. represents the estimated coefficient on the effect of each α-diversity index in the fitted random effects model

We compared EC users and non-users in each α-diversity index (i.e., Observed, Shannon [27], Simpson [28], Inverse Simpson [28], Fisher, Chao1 [29], abundance-based coverage estimator (ACE) [30], incidence-based coverage estimator (ICE) [31], phylogenetic diversity (PD) [32]) while adjusting for age, gender, and the frequency of brushing teeth in the saliva [Fig. 3A] and subgingival [Fig. 3B] sites, respectively. In the saliva site, we found significant increases in the Observed, Fisher and PD indices [32] for EC users compared to non-users [Fig. 3A]. In contrast, in the subgingival site, we found significant increases in all α-diversity indices but the Simpson index [Fig. 3B]. This may indicate that the subgingival microbiome is affected more strongly by ECs than the saliva microbiome.

We also include in the Supplementary Information [Additional file 1: Figure S1] the results of univariate analysis with no covariate adjustments. In the saliva site, we found fewer significantly different α-diversity indices in the covariate-adjusted analysis [Fig. 3A] than the analysis with no covariate adjustments [Additional file 1: Figure S1A]. In contrast, in the subgingival site, we found the same significant α-diversity indices [Fig. 3B, Additional file 1: Figure S1B]. This may indicate that age, gender, and the frequency of brushing teeth have stronger confounding effects on the disparity in microbial composition between EC users and non-users in the saliva microbiome than in the subgingival microbiome.

In addition, we assessed the disparity in α-diversity between saliva and subgingival sites from EC users [Additional file 2: Figure S2A] and non-users [Additional file 2: Figure S2B], respectively. We can see that subgingival samples have significantly higher α-diversity than saliva samples [Additional file 2: Figure S2] in both EC users and non-users. This indicates that the subgingival population tends to be more diverse than the salivary population regardless of exposure.

### β-diversity analysis

**Fig. 4:**
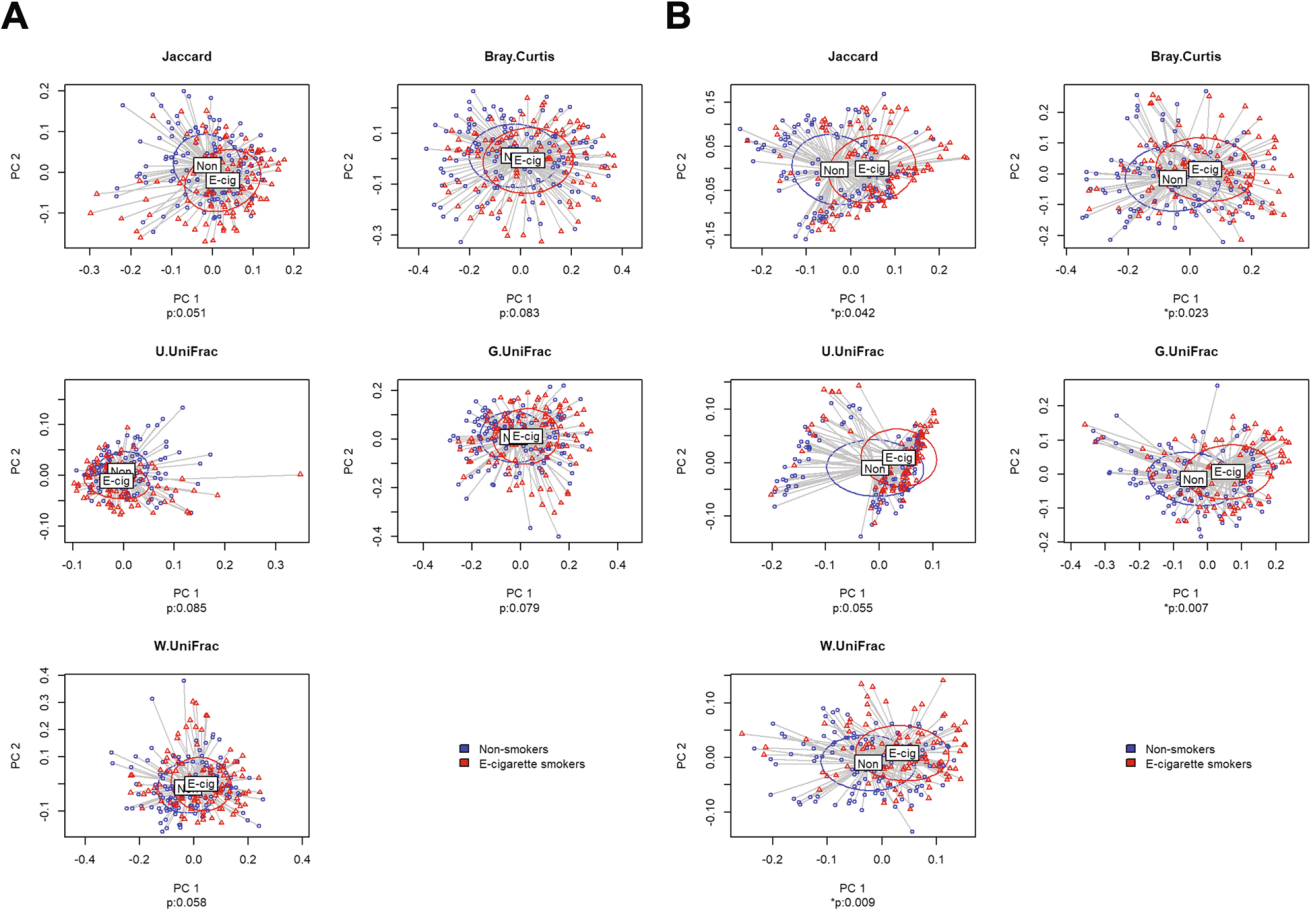
The results from β-diversity analysis. The two-dimensional PCoA plots visualize if the microbial composition is differential by the status of EC smoking with respect to each distance metric in the saliva (**A**) and subgingival (**B**) sites, respectively. *p represents the *P*-value estimated by GLMM-MiRKAT to test the disparity in each distance metric between EC users and non-users while adjusting for age, gender and the frequency of brushing teeth

Figure 4 visualizes any differentiation detected in the microbial composition between EC users and non-users with respect to each β-diversity index (i.e., Jaccard dissimilarity [33], Bray–Curtis dissimilarity [34], Unweighted UniFrac distance [35], Generalized UniFrac distance [36], and Weighted UniFrac distance [37]). The *P*-values in Fig. 4 are the ones estimated by GLMM-MiRKAT [38, 39] adjusting for age, gender, and the frequency of brushing teeth.

In the saliva site, we could not find any significant disparities between EC users and non-users in any β-diversity indices [Fig. 4A]. In contrast, in the subgingival site, we found significant disparities in all β-diversity indices but the Unweighted UniFrac distance [Fig. 4B]. Again, as in the α-diversity analysis, this may indicate that the subgingival microbiome is affected more strongly by EC use than the saliva microbiome.

We also performed and included in the Supplementary Information [Additional file 3: Figure S3] the results of univariate analysis with no covariate adjustments. As in the α-diversity analysis, the saliva site exhibited five fewer significant different β-diversity indices in the covariate-adjusted analysis [Fig. 4A] than the analysis with no covariate adjustments [Additional file 3: Figure S3A], while the subgingival site exhibited one fewer significant β-diversity index in the covariate-adjusted analysis [Fig. 4B] than the analysis with no covariate adjustments [Additional file 3: Figure S3B]. Again, this may indicate that age, gender and the frequency of brushing teeth have stronger confounding effects on the disparity in microbial composition between EC users and non-users in the saliva microbiome than in the subgingival microbiome.

## Taxonomic differential abundance analysis

**Fig. 5:**
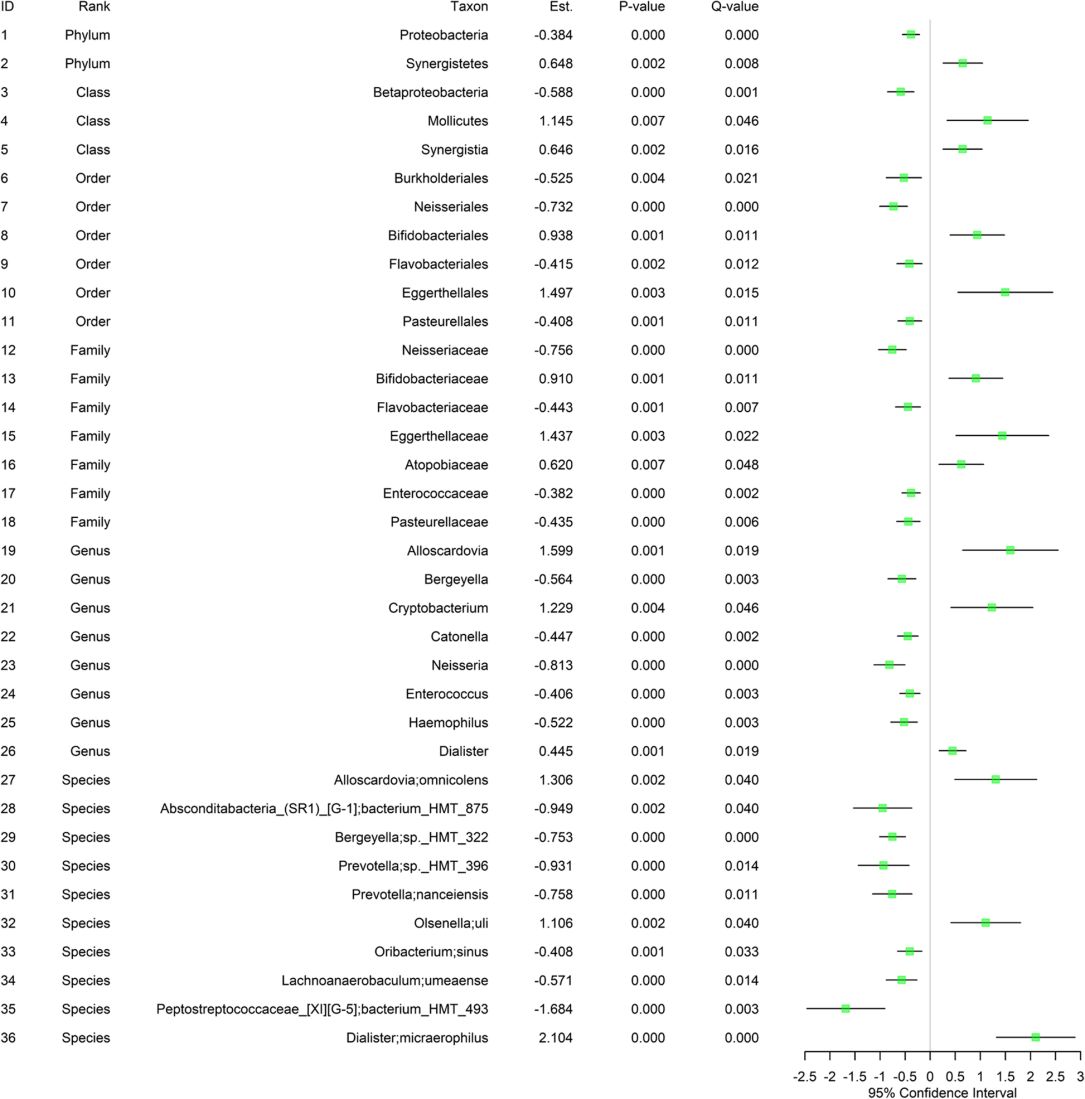
The results from taxonomic differential abundance analysis using saliva samples. The fitted random effects model to assess the disparity in each microbial taxon at each taxonomic rank (i.e., phylum, class, order, family, genus, species) between EC users and non-users while adjusting for age, gender and the frequency of brushing teeth. *Est. represents the estimated coefficient on the effect of each taxon in the fitted random effects model. *Q-value represents the *P*-value after the FDR control. *Only the statistically significant taxa after the FDR control (i.e., Q-value < 0.05) are included. For the species, the one before semi-colon (;) is the genus name and the one after semi-colon (;) is the species name

**Fig. 6:**
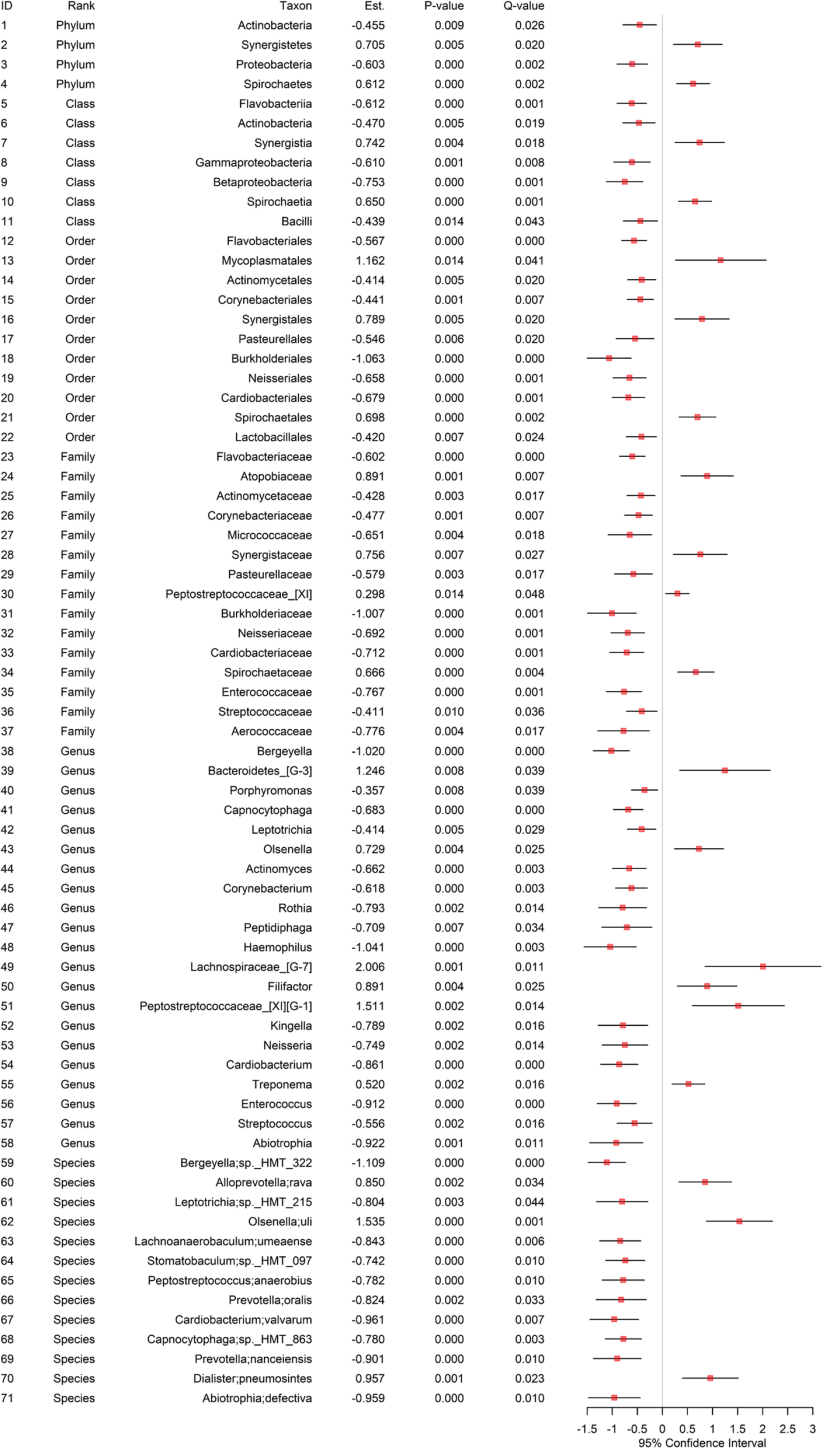
The results from taxonomic differential abundance analysis using subgingival samples. The fitted random effects model to assess the disparity in each microbial taxon at each taxonomic rank (i.e., phylum, class, order, family, genus, species) between EC users and non-users while adjusting for age, gender and the frequency of brushing teeth. *Est. represents the estimated coefficient on the effect of each taxon in the fitted random effects model. *Q-value represents the P-value after the FDR control. *Only the statistically significant taxa after the FDR control (i.e., Q-value < 0.05) are included. For the species, the one before semi-colon (;) is the genus name and the one after semi-colon (;) is the species name

Here, we assess the disparity in each microbial taxon at each taxonomic rank (i.e., phylum, class, order, family, genus, species) between EC users and non-users while adjusting for age, gender, and the frequency of brushing teeth [Fig. 5, Fig. 6]. We found significant disparities in relative abundance between EC users and non-users for: 36 microbial taxa (i.e., 2 phyla, 3 classes, 6 orders, 7 families, 8 genera, 10 species) in the saliva site [Fig. 5]; 71 microbial taxa (i.e., 4 phyla, 7 classes, 11 orders, 15 families, 21 genera and 13 species) in the subgingival site [Fig. 6]. Of these, we identified 21 microbial taxa in common (i.e., from both the saliva and subgingival sites) (i.e., 2 phyla, 2 classes, 4 orders, 5 families, 4 genera, 4 species), 15 microbial taxa (i.e., 1 class, 2 orders, 2 families, 4 genera, 6 species) only in the saliva site, and 50 microbial taxa (i.e., 2 phyla, 5 classes, 7 orders, 10 families, 17 genera, 9 species) only in the subgingival site.

**Shared by saliva and subgingival sites [Fig. ****5****, Fig. ****6****]:** A total of 21 microbial taxa were discovered in both the saliva and subgingival sites to be differentially abundant. Of these, 4 taxa showed an increase in relative abundance for EC users, while 17 microbial taxa showed a decrease in relative abundance for EC users. Interestingly, both the saliva and subgingival sites had the same effect direction (increase or decrease) for all these shared taxa in relative abundance between EC users and non-users. At the genus level, *Bergeyella*, *Neisseria*, *Enterococcus* and *Haemophilus* showed a decrease in relative abundance for EC users.

**Unique to the saliva site [Fig. ****5****]:** A total of 15 microbial taxa were discovered only in the saliva site to be differentially abundant. Of these, 10 taxa showed an increase in relative abundance for EC users, while 5 taxa showed a decrease in relative abundance for EC users. At the genus level, *Catonella* showed a decrease in relative abundance for EC users while *Alloscardovia*, *Cryptobacterium* and *Dialister* showed an increase in relative abundance for EC users.

**Unique to the subgingival site [Fig. ****6****]:** A total of 50 microbial taxa were discovered only in the subgingival site to be differentially abundant. Of these, 16 taxa showed an increase in relative abundance for EC users, while 34 taxa showed a decrease in relative abundance for EC users. At the genus level, *Bacteroidetes_[G-3]*, *Olsenella, Lachnospiraceae_[G-7]*, *Filifactor*, *Peptostreptococcaceae_[XI][G-1]* and *Treponema* showed an increase in relative abundance for EC users, while *Porphyromonas*, *Capnocytophaga*, *Leptotrichia*, *Actinomyces*, *Corynebacterium*, *Rothia*, *Peptidiphaga*, *Kingella*, *Cardiobacterium*, *Streptococcus* and *Abiotrophia* showed a decrease in relative abundance for EC users.

Overall, the greater abundance and variety of taxa cataloged at the subgingival site [Fig. 6] than at the saliva site [Fig. 5] may indicate that the oral microbiome at the subgingival site is affected more strongly or preserves microbiome changes longer than the oral microbiome at the saliva site. The difference in the taxonomic discoveries may also indicate that the oral microbiomes in the saliva and subgingival sites are affected by EC smoking in different ways.

We also include the results of univariate analysis with no covariate adjustments in the Supplementary Information [Additional file 4: Figure S4, Additional file 5: Figure S5]. We found fewer taxonomic discoveries in the analysis with covariate adjustments (i.e., a total of 36 microbial taxa) [Fig. 5] than in the analysis with no covariate adjustments (i.e., a total of 61 microbial taxa) for the saliva site [Additional file 4: Figure S4]; as such, the proportion of taxonomic discoveries in the analysis with covariate adjustments compared with the one with no covariate adjustments in the saliva site was 36/61 (i.e., about 59%). In contrast, for the subgingival site, we found relatively less fewer taxonomic discoveries in the analysis with covariate adjustments (i.e., a total of 71 taxa) [Fig. 6] than in the analysis with no covariate adjustments (i.e., a total of 82 taxa) [Additional file 5: Figure S5]; as such, the proportion of taxonomic discoveries in the analysis with covariate adjustments compared with the one with no covariate adjustments in the subgingival site was 71/82 (i.e., about 87%). As in the α-diversity and β-diversity analyses, this may indicate that age, gender, and the frequency of brushing teeth have stronger confounding effects on the disparity in microbiome composition between EC users and non-users in the saliva site.

### Mediation analysis

**Fig. 7:**
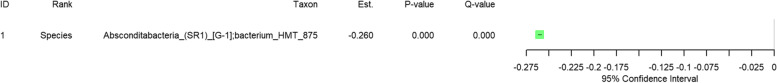
The results from mediation analysis using saliva samples. The fitted generalized linear mixed model to assess the mediating roles of the saliva microbiome between EC smoking and gingival inflammation. *Est. represents the estimated coefficient on the effect of each taxon in the fitted generalized linear mixed model. *Q-value represents the *P*-value after the FDR control. *Only the statistically significant taxa after the FDR control (i.e., Q-value < 0.05) are included. For the species, the one before semi-colon (;) is the genus name and the one after semi-colon (;) is the species name

**Fig. 8:**
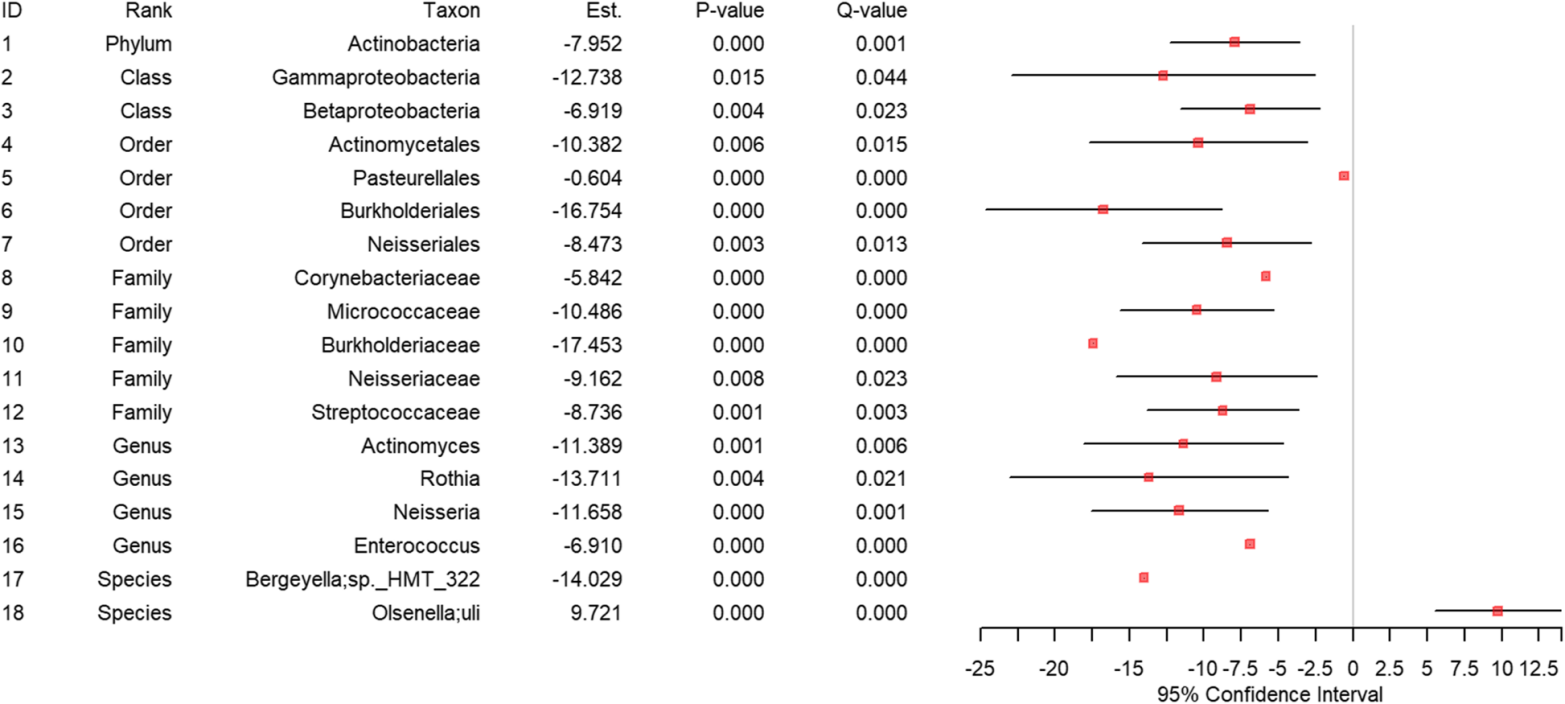
The results from mediation analysis using subgingival samples. The fitted generalized linear mixed model to assess the mediating roles of the subgingival microbiome between EC smoking and gingival inflammation. *Est. represents the estimated coefficient on the effect of each taxon in the fitted generalized linear mixed model. *Q-value represents the *P*-value after the FDR control. *Only the statistically significant taxa after the FDR control (i.e., Q-value < 0.05) are included. For the species, the one before semi-colon (;) is the genus name and the one after semi-colon (;) is the species name

To assess whether the microbial taxa that are significantly affected by ECs [Fig. 5, Fig. 6] in turn cause gingival inflammation, we fitted mediation models [26] adjusting for EC use as well as the covariates age, gender, and the frequency of brushing teeth. We identified 1 microbial taxon (i.e., 1 species) in the saliva site [Fig. 7] and 18 microbial taxa (i.e., 1 phylum, 2 classes, 4 orders, 5 families, 4 genera and 2 species) in the subgingival site [Fig. 8] as microbial taxa that mediate the effects of ECs on gingival inflammation.

In the saliva site, EC smoking significantly decreased the relative abundance of *Absconditabacteria_(SR1)_[G-1];bacterium_HMT_875* (species) [Fig. 5], and this decrease consequently resulted in gingival inflammation [Fig. 7].

In the subgingival site, EC smoking significantly decreased the relative abundance of *Actinobacteria* (phylum), *Gammaproteobacteria and Betaproteobacteria* (classes), *Actinomycetales*, *Pasteurellales*, *Burkholderiales* and *Neisseriales* (orders), *Corynebacteriaceae*, *Micrococcaceae, Burkholderiaceae, Neisseriaceae* and *Steptococcaceae* (families), *Actinomyces, Rothia, Neisseria* and *Enterococcus* (genera), and *Bergeyella;sp._HMT_322* (species) [Fig. 6], and these decreases consequently resulted in gingival inflammation [Fig. 8]. In contrast, in the subgingival site, EC smoking significantly increases the relative abundance of *Olsenella;uli*, (species) [Fig. 6], and this increase consequently resulted in gingival inflammation [Fig. 8]. Interestingly, *Olsenella;uli*, (species) is a gram-positive bacterium that is known to cause endodontic infections [40].

### Functional differential abundance analysis

**Fig. 9:**
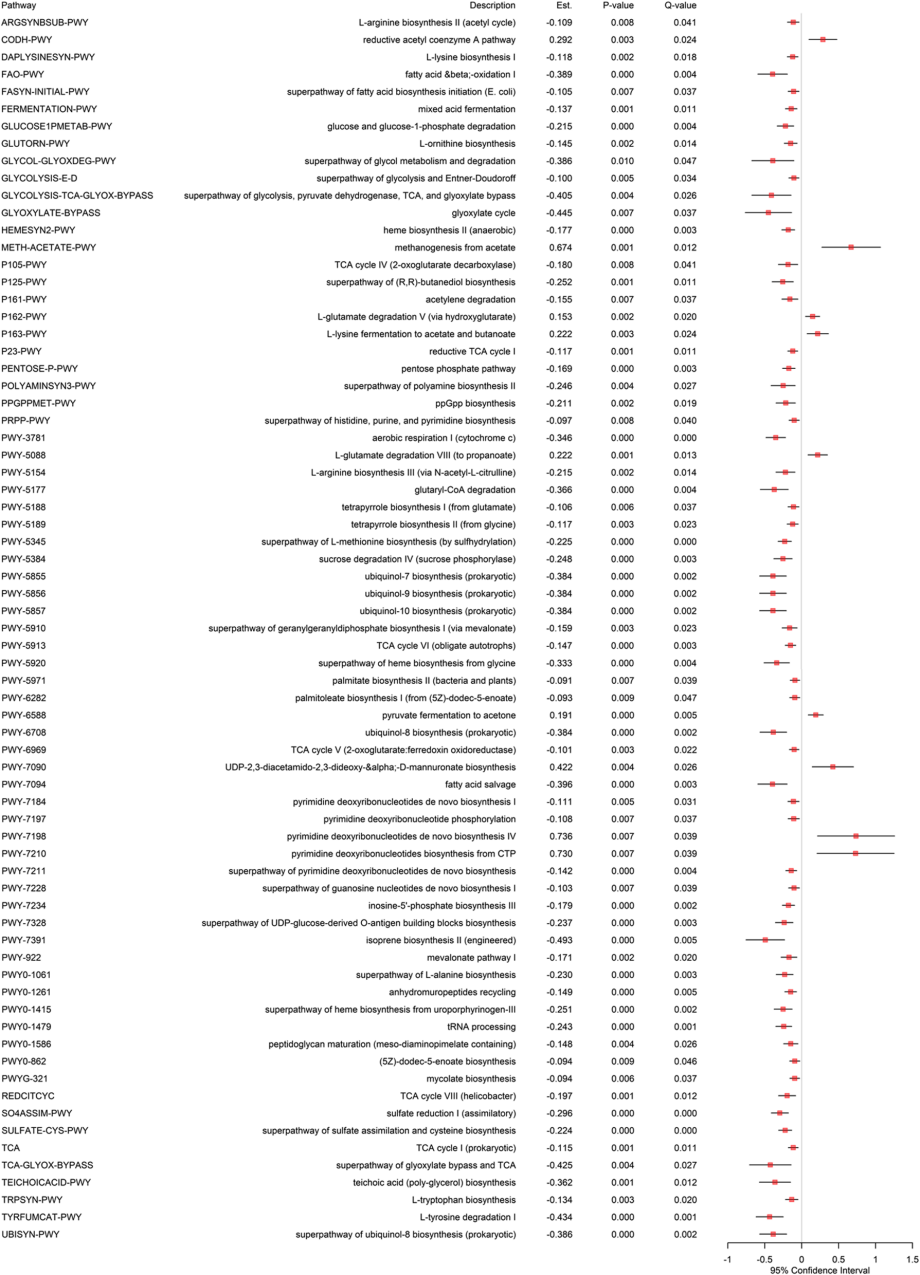
The results from functional differential abundance analysis using subgingival samples. The fitted random effects model to assess the disparity in each functional annotation (i.e., KEGG pathway) between EC users and non-users while adjusting for age, gender and the frequency of brushing teeth. *Est. represents the estimated coefficient on the effect of each pathway in the fitted effects model. *Q-value represents the *P*-value after the FDR control. *Only the statistically significant pathways after the FDR control (i.e., Q-value < 0.05) are included

We additionally assessed the disparity in each functional annotation (i.e., KEGG pathway [25]) between EC users and non-users while adjusting for age, gender, and the frequency of brushing teeth [Fig. 9]. We found significant regulations in relative abundance between EC users and non-users for 71 KEGG pathways in the subgingival site [Fig. 9], while no metabolic pathways were significantly regulated in the saliva site.

Here, we can exhibit an upregulation in metabolic pathways for obtaining energy observed in anoxic niches (e.g., fermentation) which has been confirmed in other studies of periodontal diseases [41, 42]. For example, we found an upregulation in the reductive acetyl coenzyme A pathway (CODH-PWY) [Fig. 9], which is used in anaerobic environments to fix carbon dioxide by forming acetyl-CoA that is then fermented into acetate [43]. We also detected other upregulated anaerobic metabolisms in EC users, specifically the breakdown of amino acids found in anoxic environments. The fermentation of lysine and glutamate is especially relevant to oral disease. These pathways that were observed to be upregulated in EC users are, for example, Fermentation of L-Lysine Produces Acetate and Butanoate (P163-PWY) and L-Glutamate Degradation V via hydroxylate (P162-PWY) [Fig. 9] that produce butanoate, which is an active signaling molecule in the oral cavity of the host that has been associated with inflammation, an important determinant of periodontitis [44–46]. Interestingly, our results also showed that EC users have upregulated functions observed exclusively in *Archaeans*. For example, there is increased formation of methane and carbon dioxide from acetate (acetyclastic) by upregulated anaerobic processes only observed in *Archaeans* (see methanogenesis from acetate (METH-ACETATE_PWY) [Fig. 9]) [47]. There are also synthetic pathways that are associated with increased population of these methanogens (e.g., see pyrimidine deoxyribonucleotides de novo biosynthesis IV (PWY-7198) [Fig. 9]). These results suggest that for EC users *Archaeans* are seen in combination with anaerobic fermentative bacteria in a type of symbiosis known as syntrophic association, which is represented in the functional pathways by an upregulation in both *Archaeans*-specific functions and fermentative metabolism [48–50]. In addition, we identified upregulated functional pathways that lead to the production of A-LPS, which is a specific O-antigen in the LPS from *Porphyromonas gingivalis* (e.g., see UDP-2,3-diacetamido-2,3-dideoxy-&alpha;-D-mannuronate biosynthesis (PWY-7090) [Fig. 9]), signaling the presence of this periodontal pathogen in EC users [51, 52]. Most downregulated functions were evidence of shutdown of biosynthesis in EC users (e.g., downregulated biosynthesis of amino acids, carbohydrates and aerobic processes), as there is evidence of a shift towards reductive chemistry to produce energy as described above. This is also corroborated by downregulated functions related to oxidative pathways, for example reduced TCA, glycolysis, fatty acid beta-oxidation, and aerobic respiration [Fig. 9].

## Discussion

Electronic cigarettes are a popular method of nicotine delivery, yet the long-term effects of exposure remain largely unknown. While exposure to nicotine and tobacco combustion by-products are known to alter microbial profiles of varying body sites, the capacity of electronic cigarette vapor to induce a similar change in the oral cavity is still being studied. To this aim, we sought to characterize and compare the oral microbiomes of younger adult EC users versus non-users, relating any differences to clinical evidence of disease. As outlined above, the findings from this study suggest that exposure to ECs components may cause changes to the oral microbiome at both the saliva and subgingival sites. We also observed that the use of ECs significantly increases the odds of gingival inflammation. Mediation analysis provides evidence that these changes may in turn lead to clinical evidence of gingival inflammation.

We observed a significant increase in α-diversity in the saliva and subgingival sites for EC users compared to non-users, suggesting that exposure to EC vapor increases microbial diversity. In addition, we observed a greater significant increase in α-diversity in the subgingival site compared to the saliva site, suggesting that the subgingival niche is richer in microbial diversity compared to the saliva. While we discovered more microbial taxa in the subgingival site than in the saliva site, all the discovered microbial taxa shared between the saliva and subgingival sites showed the same effect direction between EC users and non-users, suggesting that the salivary and subgingival environments are linked.

We then explored the potential underlying mechanism connecting EC smoking and gingival inflammation via the oral microbiome. To do so, we performed mediation analyses to assess whether EC smoking affects the oral microbiome, which in turn affects gingival inflammation based on the mediator and outcome models of Baron and Kenny [26]. We found evidence of microbial dysbiosis in EC users that can mediate gingival inflammation [Fig. 7 and Fig. 8]. Our results suggest that 1 microbial taxon in the saliva site [Fig. 7] and 18 microbial taxa in the subgingival site [Fig. 8] have causal mediation effects linking EC exposure to gingival inflammation. More specifically, *Absconditabacteria_(SR1)_[G-1];bacterium_HMT_875* (species) in the saliva site [Fig. 7], and *Actinobacteria* (phylum), *Gammaproteobacteria* and *Betaproteobacteria* (classes), *Actinomycetales*, *Pasteurellales*, *Burkholderiales* and *Neisseriales* (orders), *Corynebacteriaceae*, *Micrococcaceae, Burkholderiaceae, Neisseriaceae* and *Steptococcaceae* (families), *Actinomyces, Rothia, Neisseria,* and *Enterococcus* (genera), and *Bergeyella;sp._HMT_322* and *Olsenella;uli* (species) in the subgingival site [Fig. 8] appear to have the causal mediation effects. When looking at directionality, a decrease in *Absconditabacteria_(SR1)_[G-1];bacterium_HMT_875* (species) in the saliva site and a decrease in *Actinobacteria* (phylum), *Gammaproteobacteria* and *Betaproteobacteria* (classes), *Actinomycetales*, *Pasteurellales*, *Burkholderiales* and *Neisseriales* (orders), *Corynebacteriaceae*, *Micrococcaceae, Burkholderiaceae, Neisseriaceae* and *Steptococcaceae* (families), *Actinomyces, Rothia, Neisseria* and *Enterococcus* (genera) and *Bergeyella;sp._HMT_322* (species) in the subgingival site led to a greater likelihood of clinically evident gingival inflammation, while an increased in *Olsenella;uli* (species) in the subgingival site led to a greater likelihood of clinically evident gingival inflammation.

Moreover, 71 functional annotations (i.e., KEGG pathways) we discovered in the subgingival site [Fig. 9]. For example, we found upregulated pathways such as reductive acetyl coenzyme A pathway (CODH-PWY), Fermentation of L-Lysine Produces Acetate and Butanoate (P163-PWY), L-Glutamate Degradation V via hydroxylate (P162-PWY), methanogenesis from acetate (METH-ACETATE_PWY), pyrimidine deoxyribonucleotides de novo biosynthesis IV (PWY-7198), UDP-2,3-diacetamido-2,3-dideoxy-&alpha;-D-mannuronate biosynthesis (PWY-7090) [Fig. 9]). We also found downregulated functions related to oxidative pathways (see reduced TCA, glycolysis, fatty acid beta-oxidation, and aerobic respiration [Fig. 9]). These findings can provide further insights into the metabolic capacity of the oral microbiome to better understand the underlying etiology.

While previous studies have focused on salivary film, subgingival film has its own ecosystem largely due to decreased oxygen availability [53]. Various microbes that comprise this film are not easily accessible to toothbrushes, therefore subgingival samples can capture a larger unique microbial community. Interplay between microbial species within subgingival biofilms increases and fluctuates during gingival inflammation via cytokine levels [54]. When the microbial balance is disrupted in the subgingival area, it causes inflammatory exudate, specifically in the gingival crevicular fluid, leading to an increased risk of bacterial infection [55–57]. If the inflammation continues unchecked, organisms can cause localized destruction of bone and surrounding soft tissues. Conversely, inflammation from elsewhere can create an environment in which destructive pathogens are allowed to proliferate. Either pathway draws more inflammatory cells to the area, intensifying the inflammatory response. The periodontal microbial environment is critical to the health of oral ecosystems. For example, *Porphyromonas gingivalis* and *Fusobacterium nucleatum* are associated with periodontal disease progression [58]. Prevalence of *Enterococcus faecalis* in subgingival biofilm is also known to be associated with chronic periodontal infection [59]. It is possible that exposure to ECs vapor, coupled with other environmental and personal factors (e.g., diet, genetic predisposition, baseline oral microbial profile), can create conditions favorable for pathogenesis. If allowed to persist over time, these changes could lead to not only periodontal disease but also other inflammation-mediated maladies (e.g., cardiovascular disease and rheumatoid arthritis).

The discoveries described in this study have not been found in prior studies because they have primarily examined the salivary film of EC users and non-users, while not including the subgingival film. Previous studies have also focused on the differences in the taxonomic relative abundance of the oral microbial samples, while our study is the first to analyze the mediating roles of the saliva and subgingival microbial taxa in gingival inflammation. Another limitation of the current study is that study participants were not exactly matched and not all EC users were using the same EC products. Further, we only accounted for hygiene practice and gingival inflammation and did not incorporate more specific clinical markers of periodontal disease in our analysis.

## Conclusions

The results from our study provide evidence that the use of ECs is significantly associated with increases in the odds of gingival inflammation and alters the microbiome in both saliva and subgingival sites in both shared and distinct ways. To be precise, our α-diversity analysis showed that the use of ECs significantly increased Observed, Fisher and PD indices in the saliva site, while it significantly increased all α-diversity indices but the Simpson index in the subgingival site. In addition, we observed a significantly higher α-diversity in the subgingival site compared to the saliva site for both EC users and non-users. Our β-diversity analysis showed that the use of ECs did not change any β-diversity index in the saliva site, while it changed all β-diversity indices but the Unweighted UniFrac distance in the subgingival site. Thus, both of our α-diversity and β-diversity analyses indicated that the subgingival microbiome is affected more strongly by EC use than the saliva microbiome.

Our taxonomic differential abundance analysis found significant disparities in relative abundance between EC users and non-users for 36 microbial taxa in the saliva site and 71 microbial taxa in the subgingival site. This also indicates that the subgingival microbiome is affected more strongly by ECs than the saliva microbiome. We identified 21 microbial taxa in both the saliva and subgingival sites, and they showed the same effect direction (increase or decrease) from non-users to EC users. These findings are consistent with other studies reporting an association between increased microbial richness and periodontitis [60]. Furthermore, our mediation analyses revealed that 1 microbial taxon in the saliva site and 18 microbial taxa in the subgingival site [Fig. 8] have causal mediation effects linking EC exposure to gingival inflammation. In addition, we discovered 71 significantly differential functional annotations (i.e., KEGG pathways) in the subgingival site, but no ones in the saliva site. Our functional differential abundance analysis revealed that subgingival samples from EC users exhibited an increase in metabolic pathways for obtaining energy observed in anoxic niches (e.g., fermentation) and increased anaerobic metabolism in EC users. Interestingly, our results also showed that EC users have increased functions observed exclusively in *Archaeans* that are in combination with anaerobic fermentative bacteria in a type of symbiosis known as syntrophic association, which is represented in the functional pathways by an increase of both *Archaeans*-specific functions and fermentative metabolism [48–50]. In addition, we identified increased functional pathways that lead to the production of A-LPS, which is a specific O-antigen in the LPS from *Porphyromonas gingivalis*, signaling the presence of this periodontal pathogen in EC users [51, 52]. Most downregulated functions were evidence of shutdown of biosynthesis in EC users (e.g., decreased biosynthesis of amino acids, carbohydrates and decreased aerobic processes).

Overall, although our study did not include teenagers, who compose the most at-risk group for EC related issues largely in part to their tobacco naivety, it did assess the effects of ECs on younger adults, most of whom are daily users of these products. Findings from this study suggest that exposure to ECs may cause changes to both the saliva and subgingival microbial environments that may in turn have a clinical consequence (i.e., gingival inflammation). If allowed to persist, these early changes may be a harbinger of other later maladies such as periodontitis, edentulism and cancer. Although it is unclear whether discontinuation of EC use could result in a regulation of the oral microbiome**,** the potential health effects of continued exposure through continued use is of concern. It is also important to note that exposure to tobacco smoke, alcohol, and having ‘poor oral health’ are all associated with an increased risk of HPV-related oropharyngeal squamous cell carcinoma. While studies to elucidate this relationship are ongoing, one proposed mechanism is that persistent inflammation in the setting of oral HPV infection may prevent viral clearance, leading to more chances of replication errors that evade the host immune response. Theoretically, a similar mechanism could be at play with electronic cigarette exposure. Additional research is required on this topic.

Our findings also suggest that continued surveillance of the health effects of electronic cigarette exposure is required. At minimum, our study highlights the need for additional research regarding the microbial and clinical effects of e-cigarette exposure on the body.

## Methods

### Participants and recruitment

We recruited participants from Baltimore, MD and its surrounding areas, advertising through social media (e.g., Facebook and Instagram), daily newspapers (e.g. Baltimore Sun), and flyers posted at local vape shops and colleges. Eligibility screening was conducted via phone calls prior to the participant’s first visit. All participants were between 18 and 34 years in age and did not use any tobacco products in the last three months prior to enrollment (and throughout the study) and had less than 5 pack-years of exposure to tobacco products. Participants were excluded from the study if they received any dental cleaning within the last 3 months from the baseline visit. In addition, participants did not have any antibiotics or oral/inhaled corticosteroids use for at least 3 months prior to their baseline visit. EC users were defined as individuals who have been smoking ECs that contain nicotine daily for at least 6 months and having a positive urine cotinine test result at their baseline visit. Non-users were defined as individuals who have not used EC products for at least 90 days and had a negative urine cotinine test result at their baseline visit. The VAPORS study was designed as a longitudinal observational study, consisting of 3 visits over a 2-year span. However, we couldn’t collect the microbiome samples from the last visit due to the outbreak of the COVID-19 pandemic, but we were able to process the first cross-sectional datasets and partial second visit samples. The first study visits were completed between June 2017 and October 2018. A total of 150 participants were enrolled, consisting of 75 EC users and 75 non-users. All participants provided informed consent and received financial compensation.

### Sample collection and processing

Prior to arrival for the visit, participants were instructed to refrain from the following activities for a minimum of 8 h: eating, tooth brushing, flossing, rinsing, and gum chewing. Saliva samples were collected with the supervision of the study coordinator via the passive drool method using a disposable Saliva Collection Aid (Salimetrics #5016.02) placing it into a cryovial. Saliva samples were then aliquoted for analysis of the microbiome and quantification of analytes. The study dentist, Leah Leinbach, and other qualified dental residents completed the oral exams and subgingival plaque collections. The oral exam evaluated the participant’s extra- and intraoral structures. Subgingival plaques were collected by inserting endodontic paper points (Spident # 104–0245) into the interproximal gingival sulci of 10 randomly selected teeth until tissue resistance, using a sterile dental tweezer. Each paper point was left in place for 10 s to allow absorption of crevicular fluid containing bacteria, then removed, pooled, and placed in a buffer and frozen at -80 °C until microbiome profiling was conducted.

### Gingival inflammation

Gingival inflammation was determined by the study dentist, Leah Leinbach, using a scale of 0 – 3 (0 = normal gingiva; 1 = mild inflammation; 2 = moderate inflammation; 3 = severe inflammation). This scale was reviewed with dental clinicians. To account for potential interobserver bias, gingival inflammation was further dichotomized into either no inflammation (0 = absence; normal gingiva) (i.e., clinical health) and inflammation (1 = presence; mild, moderate or severe inflammation) (i.e., evidence of erythema, edema, etc.).

### Extraction of nucleic acids

DNA was isolated using a Zymo Research Quick-DNA Fecal/Soil Microbe Miniprep Kit (catalog D6010) from paired saliva and subgingival plaque samples. Saliva samples were stored as a 1:1 ratio of saliva to *RNALater*. The tips of 20 subgingival plaque sample paper points were cut and placed in a bead tube containing 800 µl GeneJET Genomic DNA Purification Kit (R1511) lysis buffer. Following the manufacturer’s protocol, 750 µl saliva and 800 µl subgingival lysates were disrupted using an Omni Bead Ruptor 12 for 3 cycles of 30 s at 6 m/s with 5 min of dwelling on ice between cycles, with the exception that the subgingival plaque samples were disrupted in the provided bead tubes. Concentration was measured on a Qubit 4 Fluorometer using a dsDNA BR Kit (ThermoFisher) and DNA was diluted to 5 ng/µl for library preparation. Libraries were made following Illumina protocol 16S V3-V4-metagenomic-library-prep-guide-15044223-b using the gene-specific amplicon primer sequence provided and Nextera XT Index kits (catalog 15,052,163–6). The V3-V4 amplicon primers were synthesized using Integrated DNA Technologies. The V3-V4 PCR primer sequences were 16SAmpliconPCRForwardPrimer = 5’TCGTCGGCAGCGTCAGATGTGTATAAGAGACAGCCTACGGGNGGCWGCAG and 16SAmpliconPCRReversePrimer = 5'GTCTCGTGGGCTCGGAGATGTGTATAAGAGACAGGACTACHVGGGTATCTAATCC. Library concentrations were measured on a Qubit 4 Fluorometer using a dsDNA BR Kit (ThermoFisher). Library quality and size was validated on an Agilent Fragment Analyzer using a DNF-467-Genomic-DNA-50 Kb-Analysis-Kit. Libraries were pooled at 5 nM using Qubit concentrations.

### Metagenomic sequencing and quality control

We conducted raw sequence data processing, denoising and taxonomic annotations using the pipeline, QIIME2 [61] and the expanded human oral microbiome database (eHOMD) (16S rRNA RefSeq version 15.22) [62]. We also used DADA2 and USEARCH to remove sequencing errors and chimeras. We used PICRUSt2 to generate functional annotations (i.e., KEGG pathways) [63]. The microbiome data initially had 19,607 amplicon sequence variants (ASVs) for 426 samples (225 saliva and 201 subgingival samples). We applied quality control criteria to keep the samples that have library sizes larger than 5,000 and ASVs that have mean proportions larger than 2x ${10}^{5}$. As a result, 2,323 ASVs for 426 samples (225 saliva and 201 subgingival samples) were finally retained in our analysis.

### α-diversity analysis

We calculated nine α-diversity indices (i.e., Observed, Shannon [27], Simpson [28], Inverse Simpson [28], Fisher, Chao1 [29], ACE [30], ICE [31], PD [32]) using the R packages aMiAD [64], fossil [65], picante [66] and entropart [67]. We fitted the random effects model [68] to assess the disparity in each α-diversity index between EC users and non-users while adjusting for age, gender and the frequency of brushing teeth. For paired microbiome analysis, we created box-plots to visualize subgingival and saliva samples for EC users and non-users, respectively, in each α-diversity index. We used the Wilcoxon signed-rank test [69] to estimate the disparity in each α-diversity index between subgingival and saliva sites.

### β-diversity analysis

We calculated five ecological distance metrics (i.e., Jaccard dissimilarity [33], Bray–Curtis dissimilarity [34], Unweighted UniFrac distance [34], Generalized UniFrac distance (θ = 0.5) [34], and Weighted UniFrac distance [34]) using the R packages, GUniFrac [36] and MiRKAT [38, 39]. We created two-dimensional PCoA plots to visualize each of the five ecological distance metrics stratified by EC smoking status. We used GLMM-MiRKAT [70] to estimate the disparity in each distance metric between EC users and non-users while adjusting for age, gender, and the frequency of brushing teeth.

### Taxonomic differential abundance analysis

We applied the centered log-ratio (CLR) transformation [71] using the R package, compositions, to relax the compositional constraint. We fitted the random effects model [68] to assess the disparity in each microbial taxon at each taxonomic rank (i.e., phylum, class, order, family, genus, species) by EC smoking status while adjusting for age, gender and the frequency of brushing teeth. We applied the Benjamini-Hochberg (BH) procedure [72] per taxonomic rank to set a false discovery rate (FDR) threshold of under 5%.

### Functional differential abundance analysis

We applied the CLR transformation [71] using the R package, compositions, to relax the compositional constraint. We fitted the random effects model [68] to assess the disparity in each pathway by EC smoking status while adjusting for age, gender and the frequency of brushing teeth. We applied the BH procedure [72] per taxonomic rank to set a control FDR threshold of under 5%.

### Mediation analysis

To assess the mediating roles of the oral microbiome between EC smoking and gingival inflammation, we followed the Baron and Kenny’s procedures [26]. That is, we first fitted the mediator models to assess the disparity in microbial taxa by gingival inflammation status adjusting for age, gender and the frequency of brushing teeth, which was done in *Taxonomic differential abundance analysis*. Then, we fitted the outcome models using the generalized linear mixed model [73] to assess the effects of the significantly differential taxa in the mediator models on the gingival inflammation while adjusting for EC smoking status as well as the other covariates, age, gender, and the frequency of brushing teeth. Here, the adjustment of EC smoking in the outcome models was necessary to exclude possible direct effect of EC smoking on gingival inflammation, which is not transmitted by the oral microbiome [26]. As in *Taxonomic differential abundance analysis*, we applied the CLR transformation [71] to relax the compositional constraint using the R package, compositions. We applied the BH procedure per taxonomic rank to set an FDR threshold of under 5%.

## Supplementary Information

Below is the link to the electronic supplementary material.

**Additional file 1: Figure S1.** The results from unadjusted α-diversity analysis using saliva (A) and subgingival (B) samples.

**Additional file 2: Figure S2.** The results from α-diversity analysis for EC users (A) and non-users (B).

**Additional file 3: Figure S3.** The results from unadjusted β-diversity analysis using saliva (A) and subgingival (B) samples.

**Additional file 4: Figure S4.** The results from unadjusted taxonomic differential abundance analysis using saliva samples.

**Additional file 5: Figure S5.** The results from unadjusted taxonomic differential abundance analysis using subgingival samples.

## Data Availability

Sequencing data from this study have been submitted to the NCBI Gene Expression Omnibus (GEO; http://www.ncbi.nlm.nih.gov/geo/) under accession number GSE201949.

## Abbreviations

ACE: Abundance-based coverage estimator
ASV: Amplicon sequence variant
BH: Benjamini-hochberg
CLR: Centered log-ratio
EC: Electronic cigarettes
FDR: False discovery rate
GI: Gastrointestinal tract
GU: Genitourinary tract
ICE: Incidence-based coverage estimator
KEGG: Kyoto encyclopedia of genes and genomes
MiRKAT: Microbiome regression-based kernel association test
OR: Odds ratio
PCoA: Principal coordinate analysis
PD: Phylogenetic diversity
PE: Phylogenetic entropy
PQE: Phylogenetic quadratic entropy
UniFrac: Unique fraction
