# Supplementary figures and images for: The mediating roles of the oral microbiome in saliva and subgingival sites between e-cigarette smoking and gingival inflammation
Source: BMC Microbiol. 2023 Feb 2;23:35. doi: 10.1186/s12866-023-02779-z (PMC9893987; doi:10.1186/s12866-023-02779-z)

A

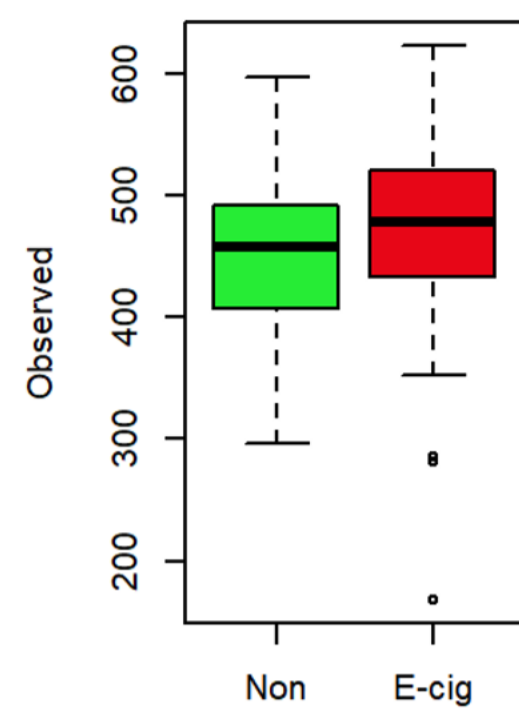

\*p:0.011

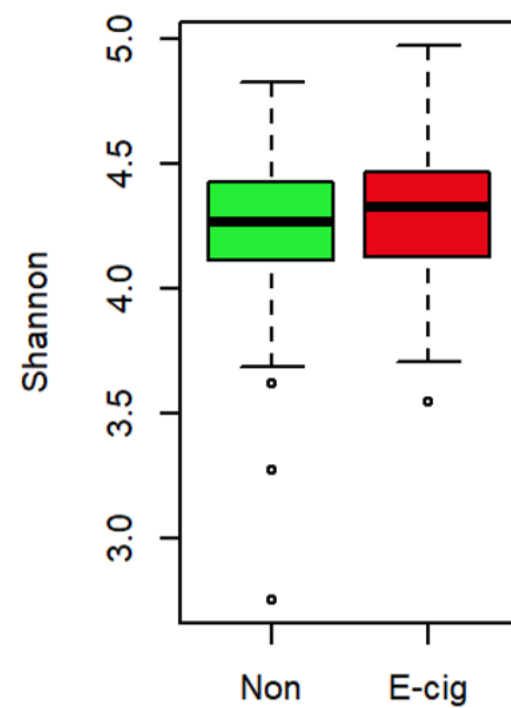

p:0.228

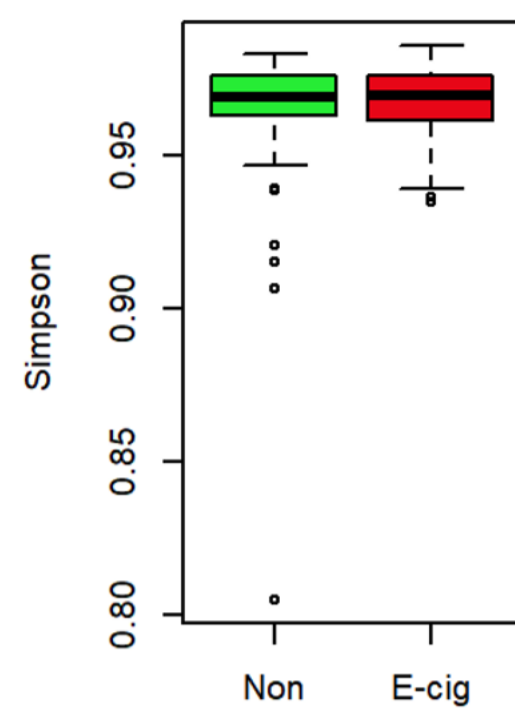

p:0.5

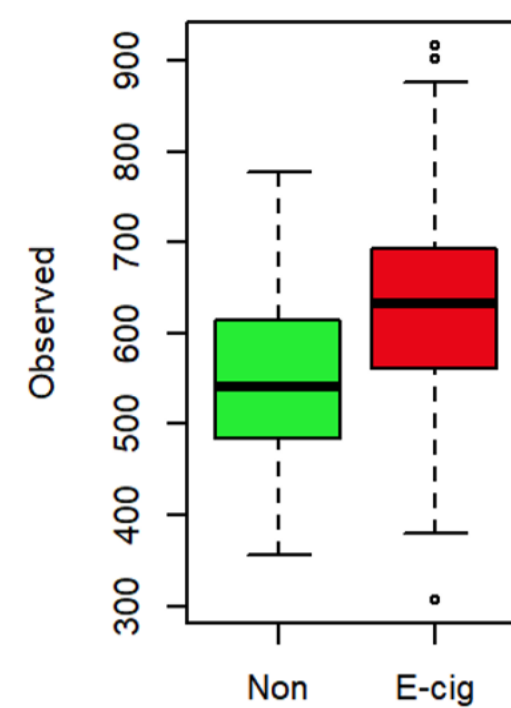

\*p:&lt;.001

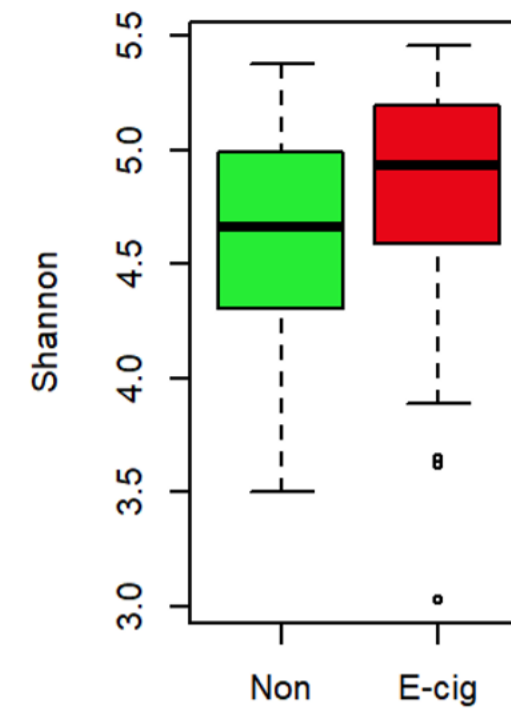

\*p:0.001

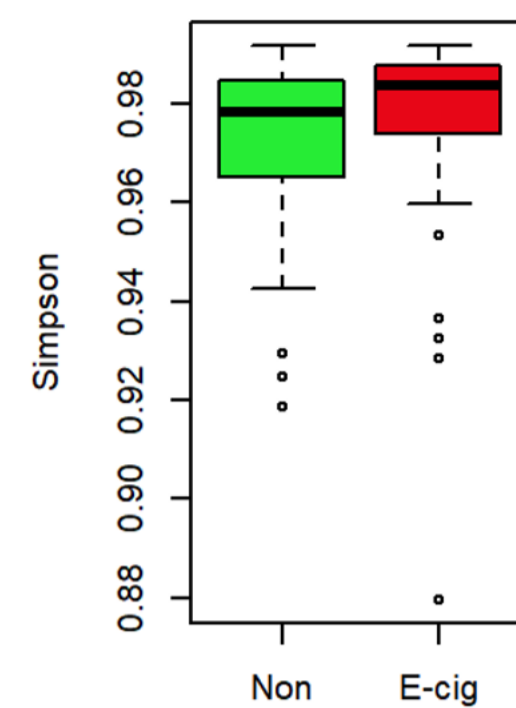

p:0.087

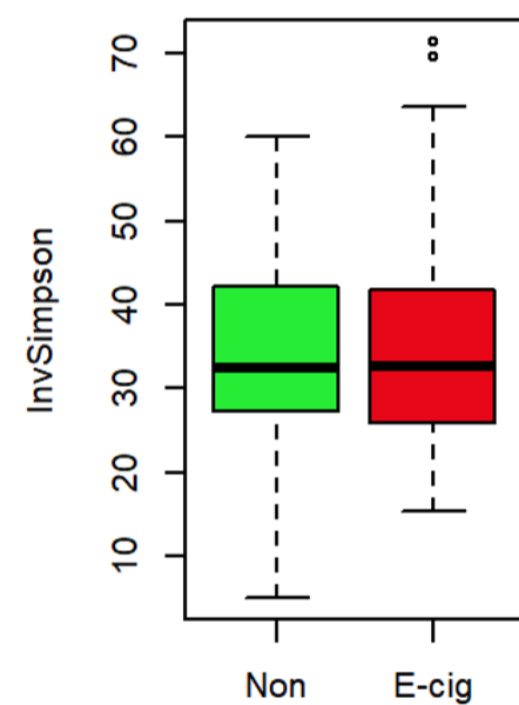

p:0.754

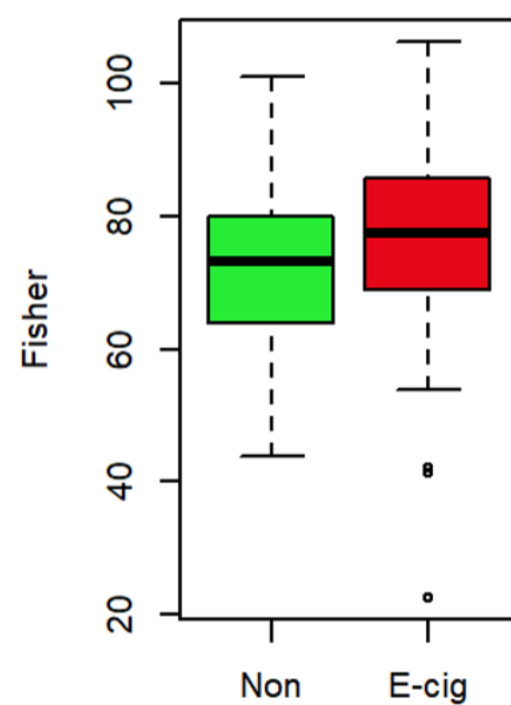

\*p:0.009

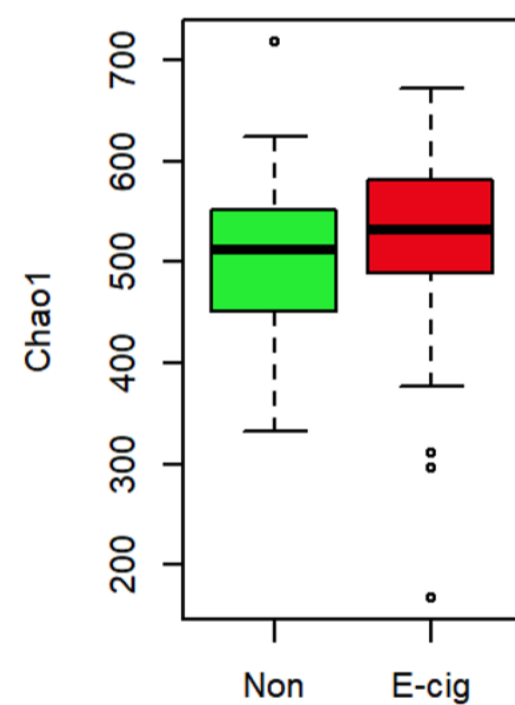

p:0.08

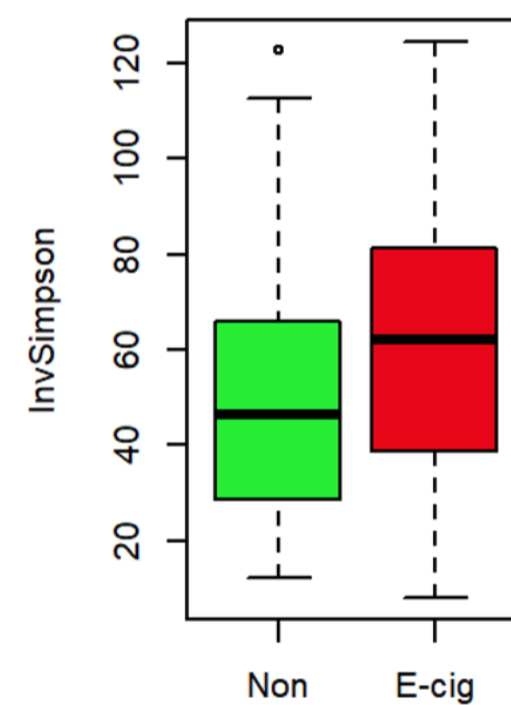

\*p:0.008

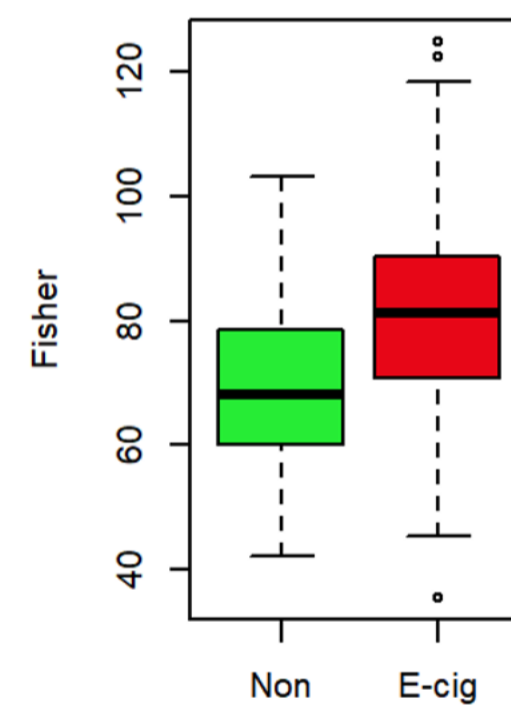

\*p:&lt;.001

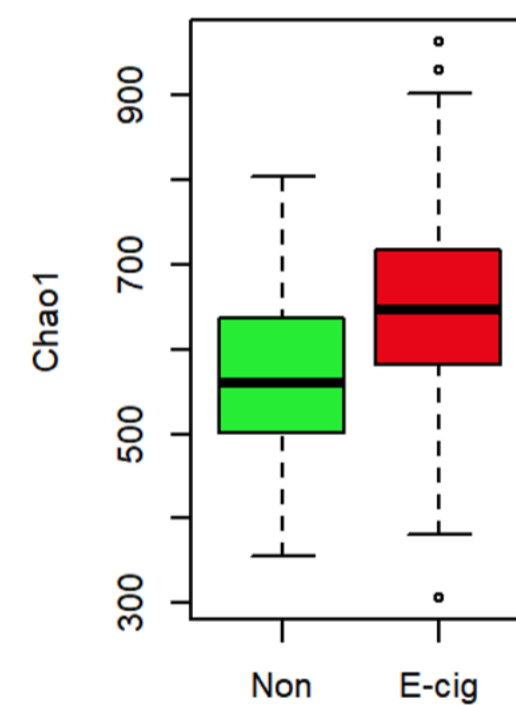

\*p:&lt;.001

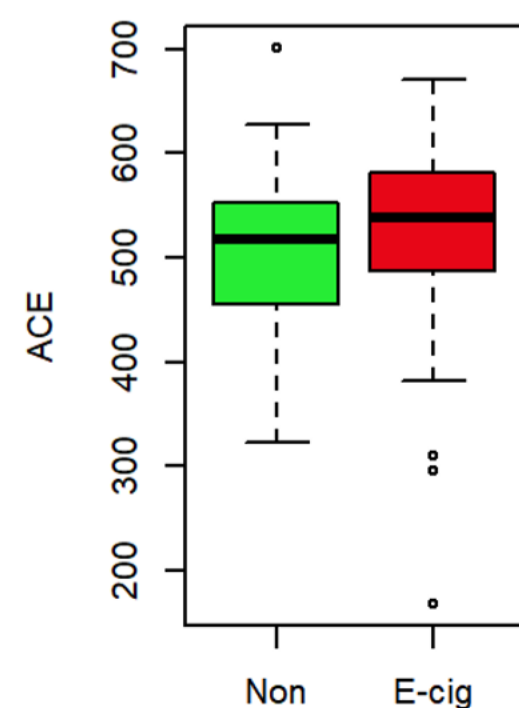

p:0.092

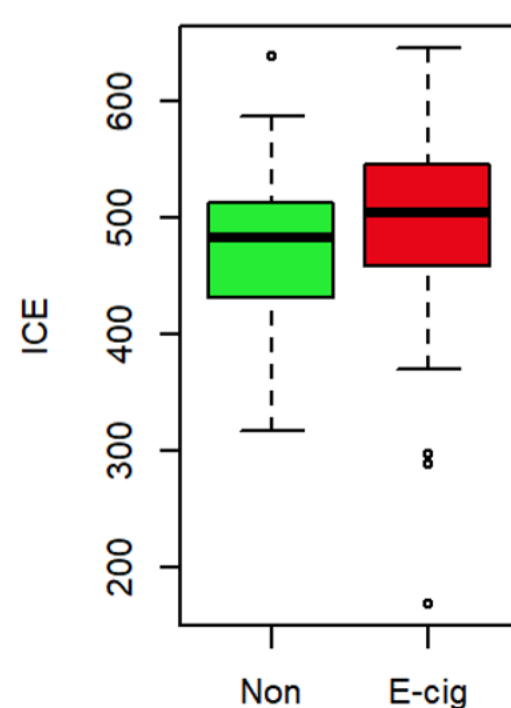

\*p:0.027

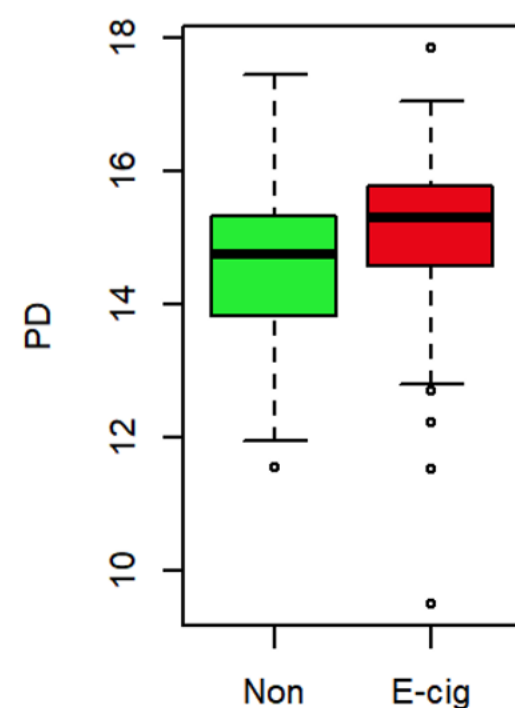

\*p:0.004

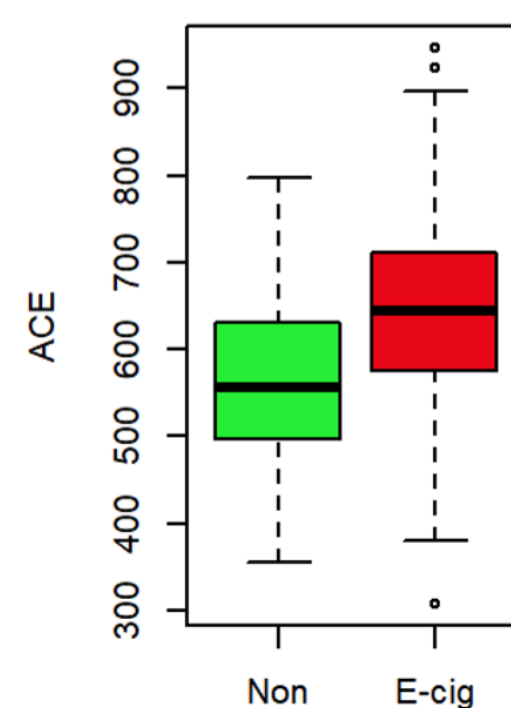

\*p:&lt;.001

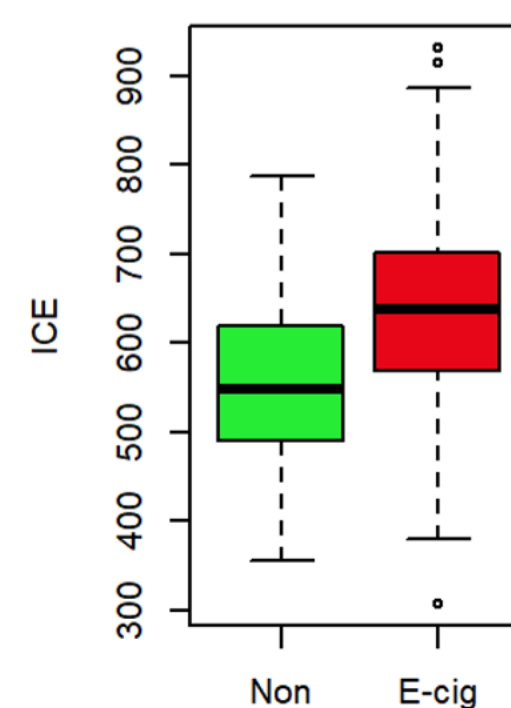

\*p:&lt;.001

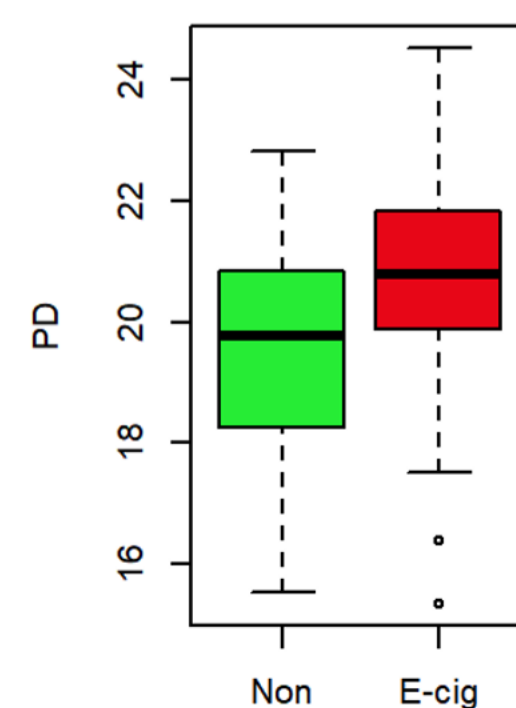

\*p:&lt;.001

B

Supplement: Supplementary file 1 — Additional file 1: Figure S1. The results from unadjusted α-diversity analysis using saliva (A) and subgingival (B) samples. [file 12866_2023_2779_MOESM1_ESM.pdf]

**A**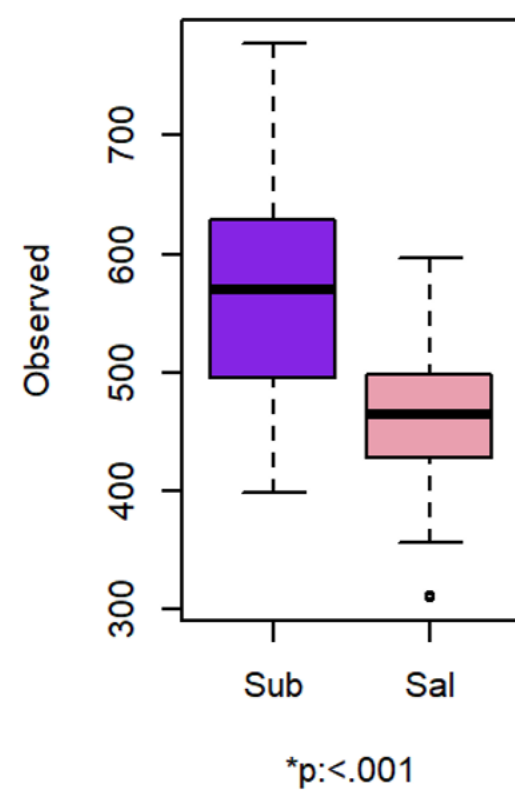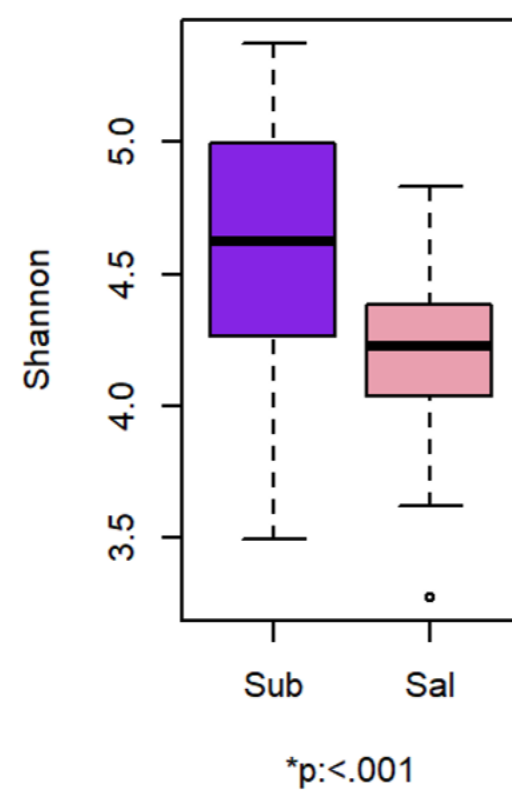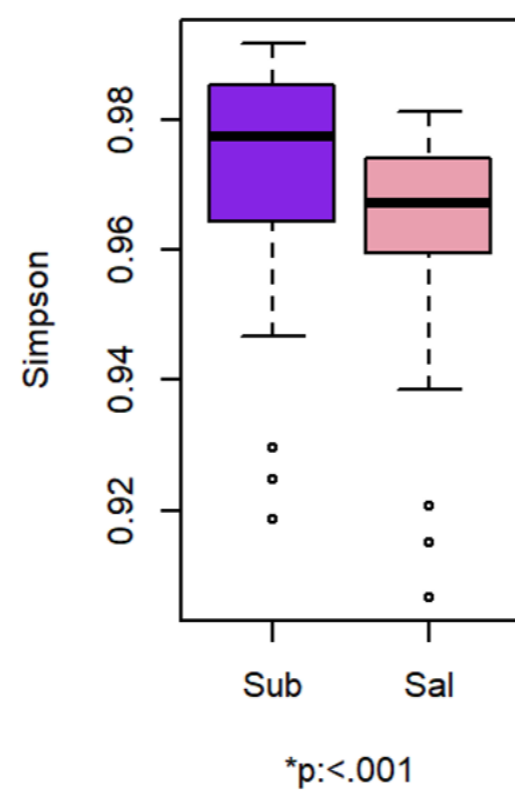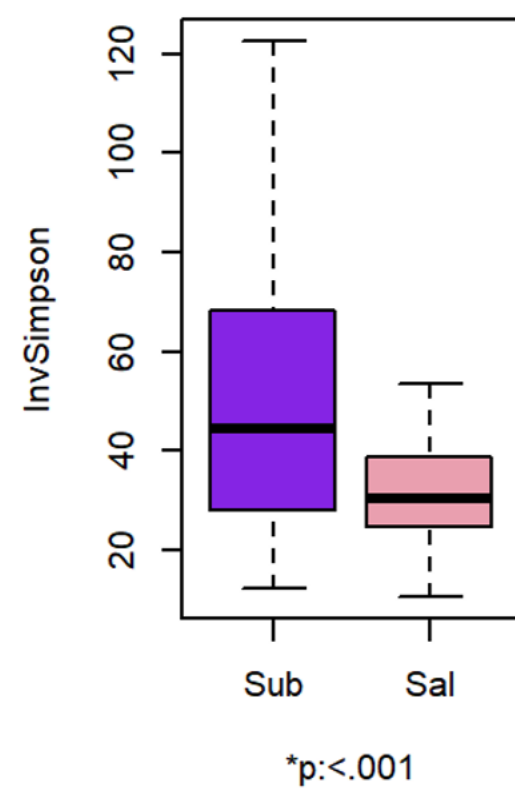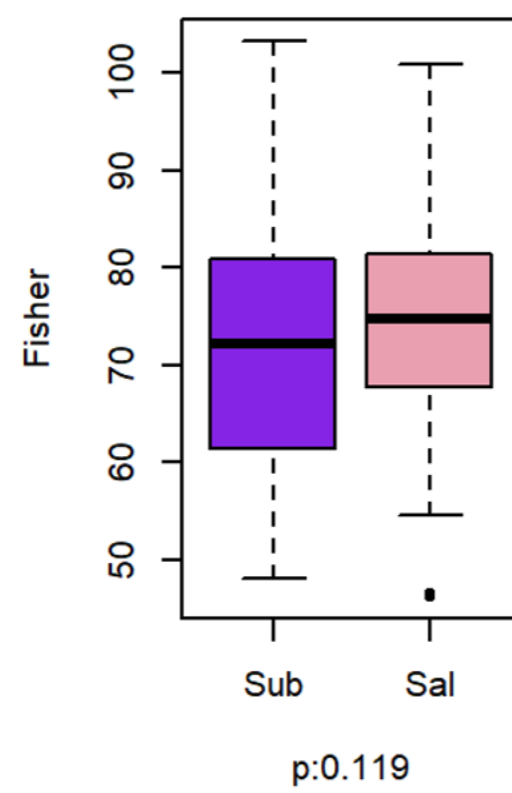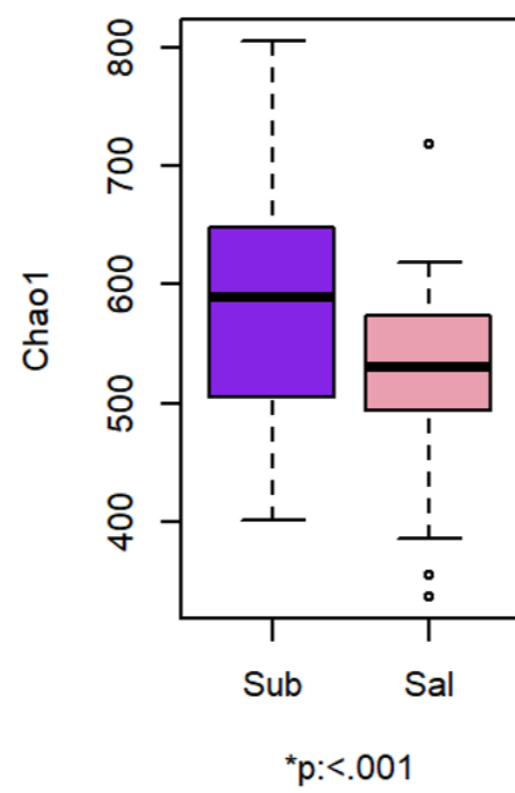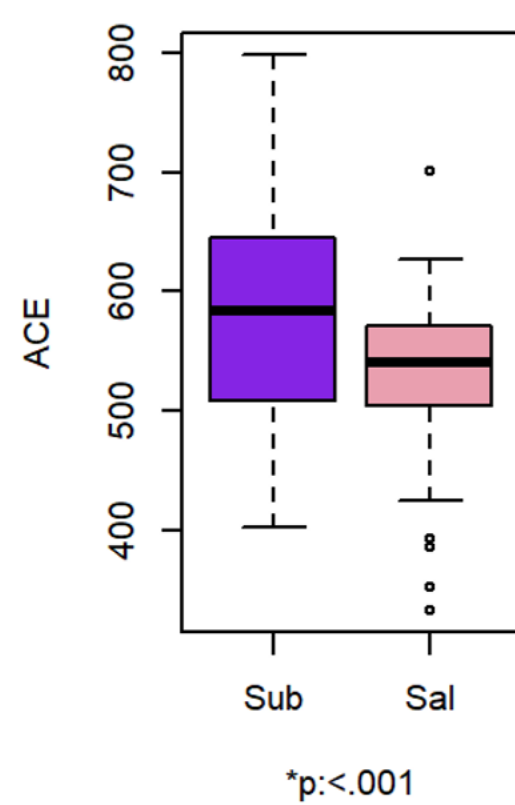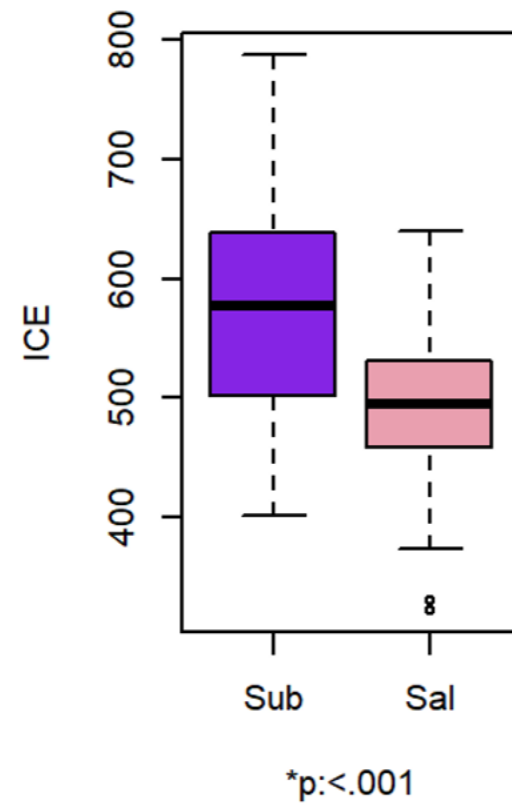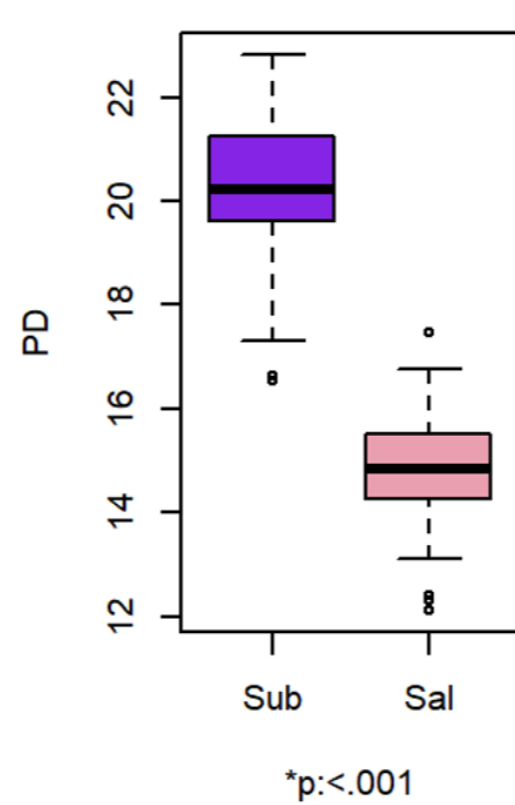**B**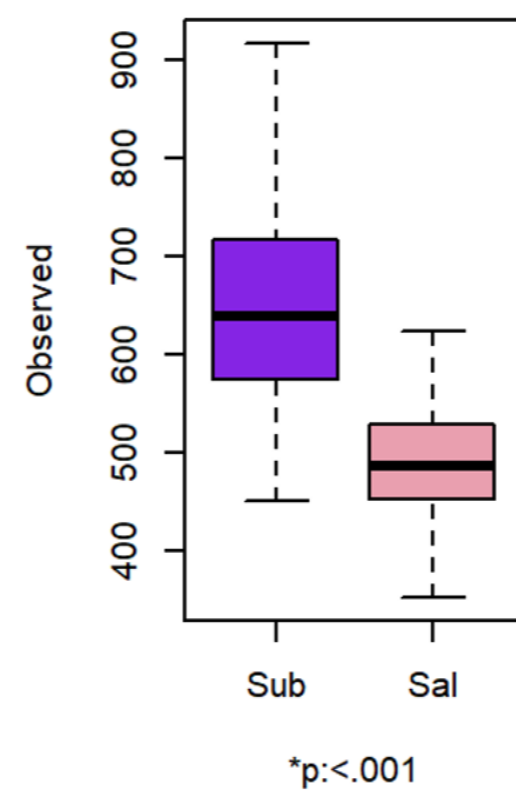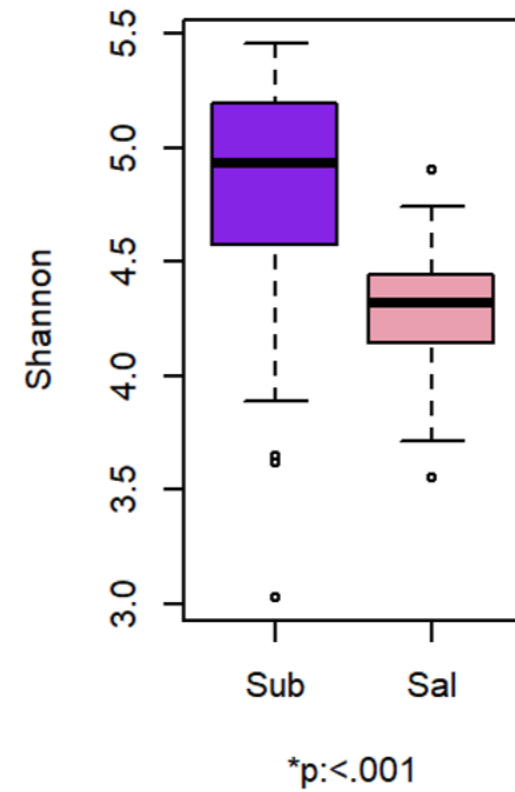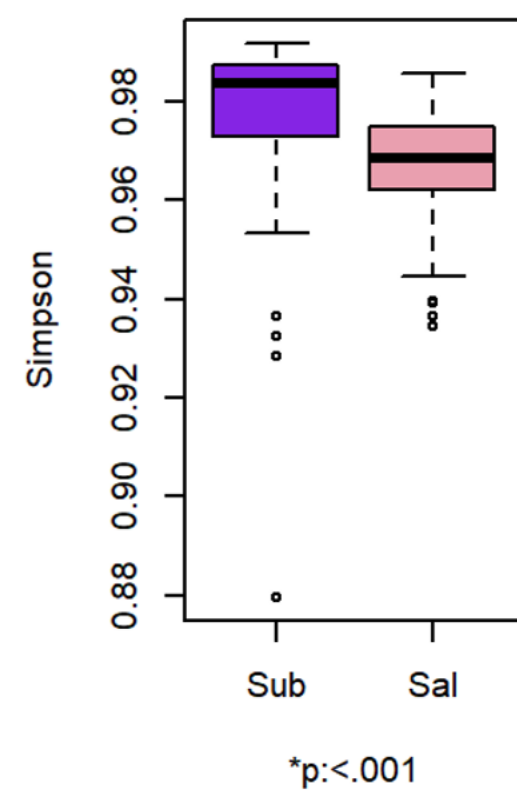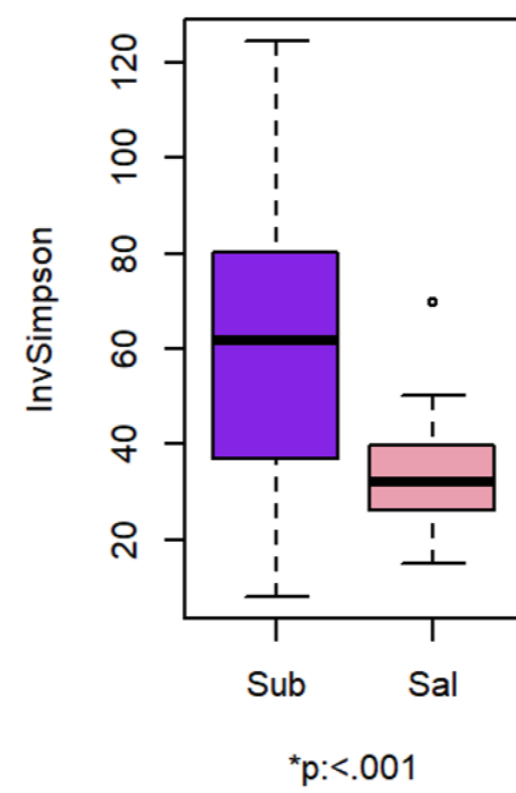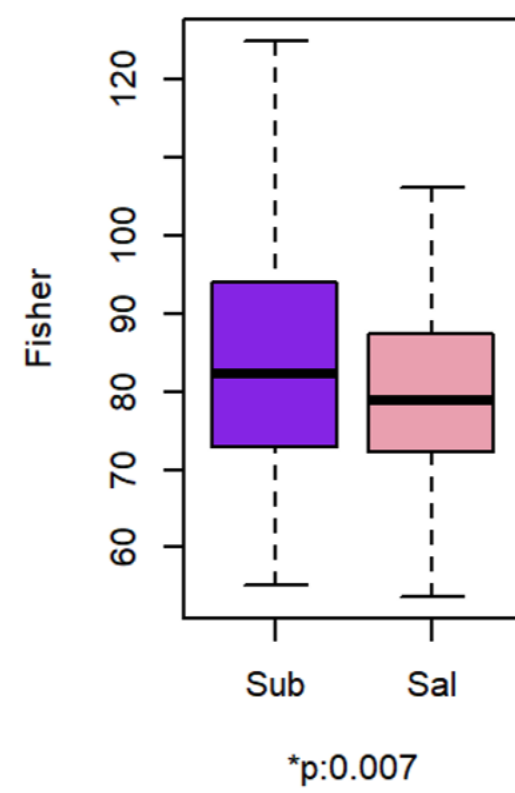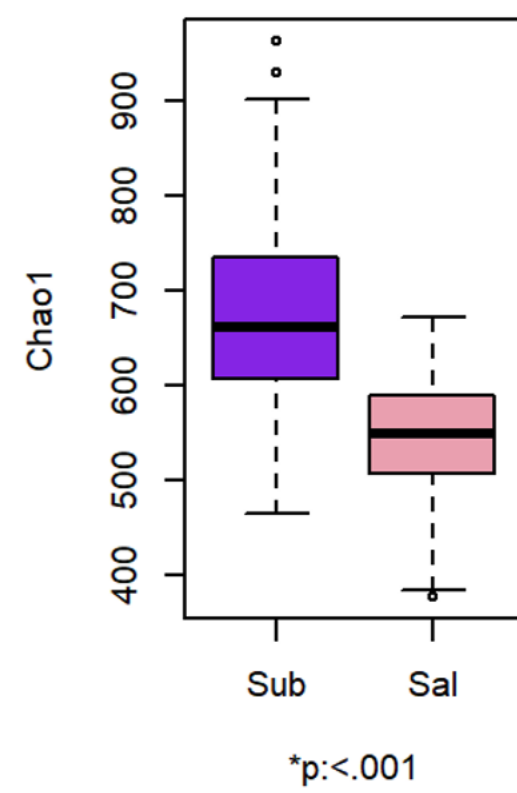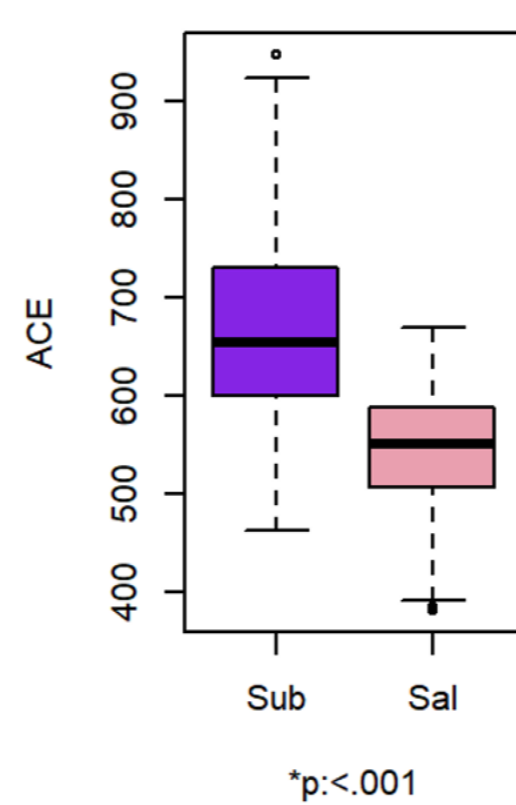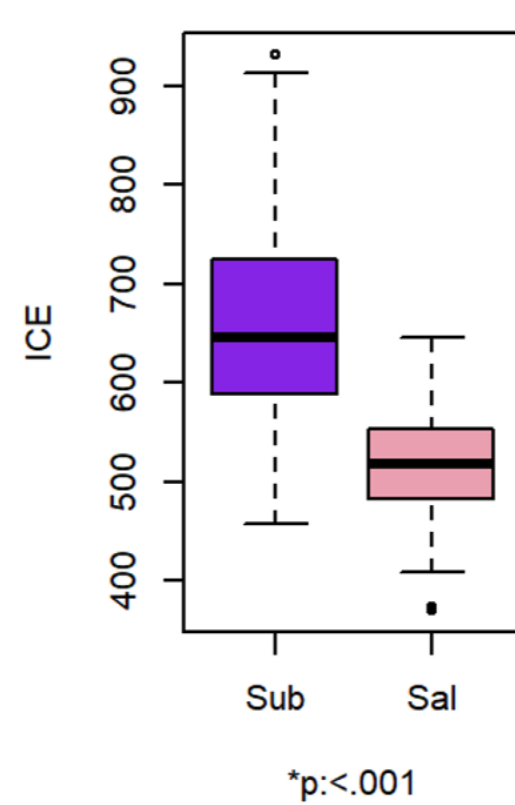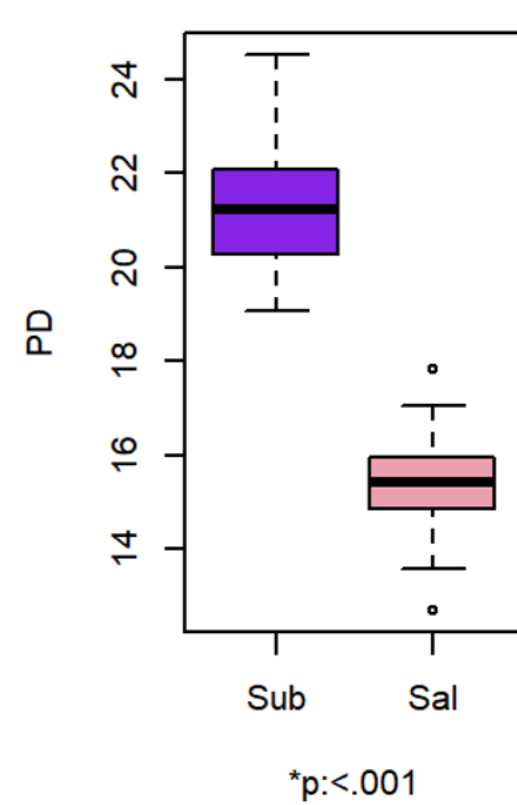

Supplement: Supplementary file 2 — Additional file 2: Figure S2. The results from α-diversity analysis for EC users (A) and non-users (B). [file 12866_2023_2779_MOESM2_ESM.pdf]

**A**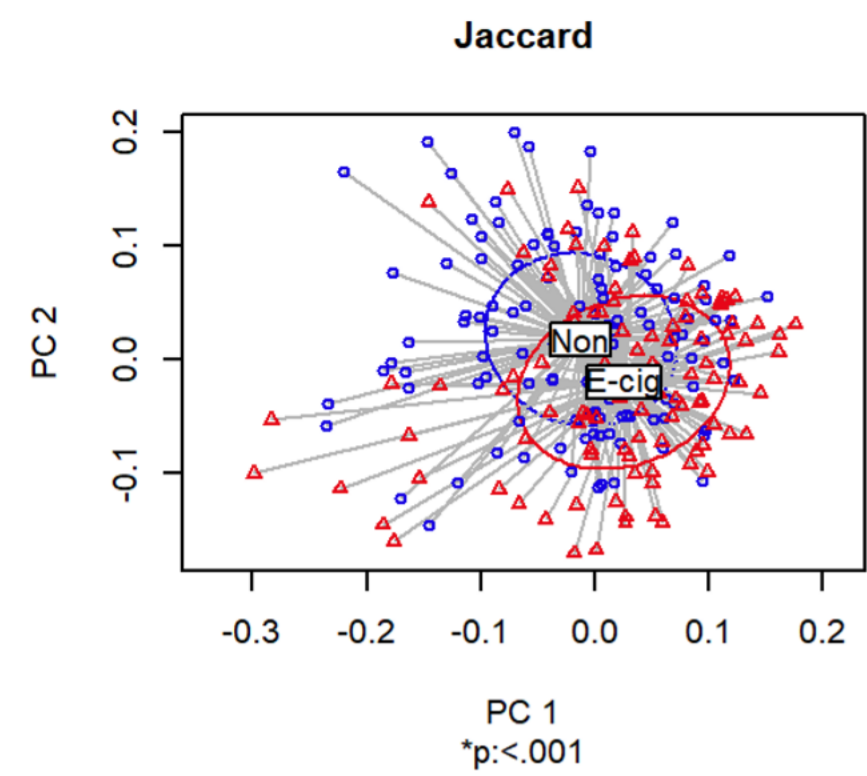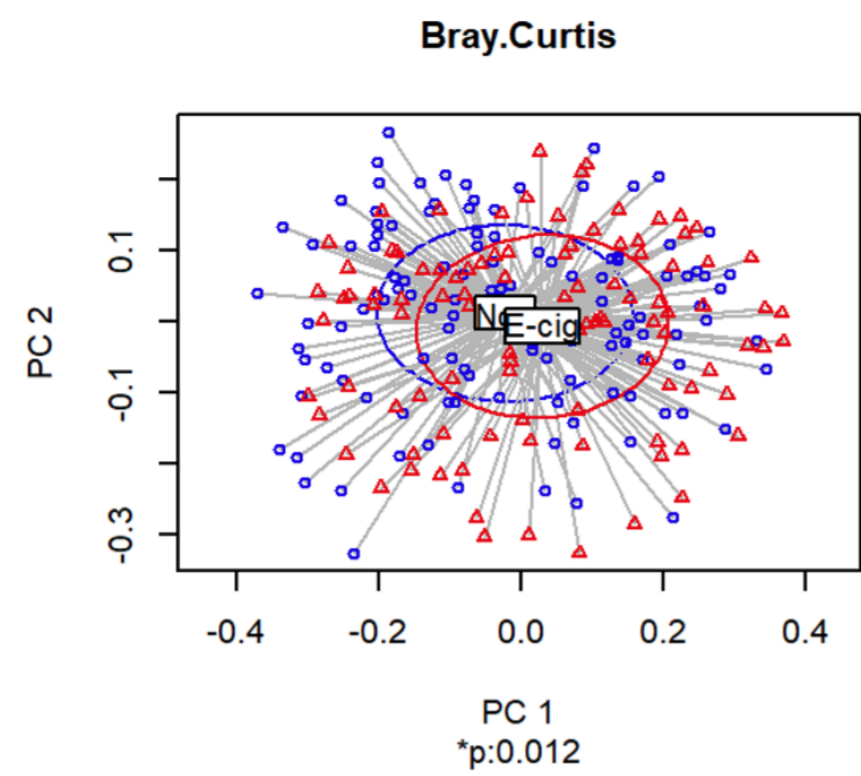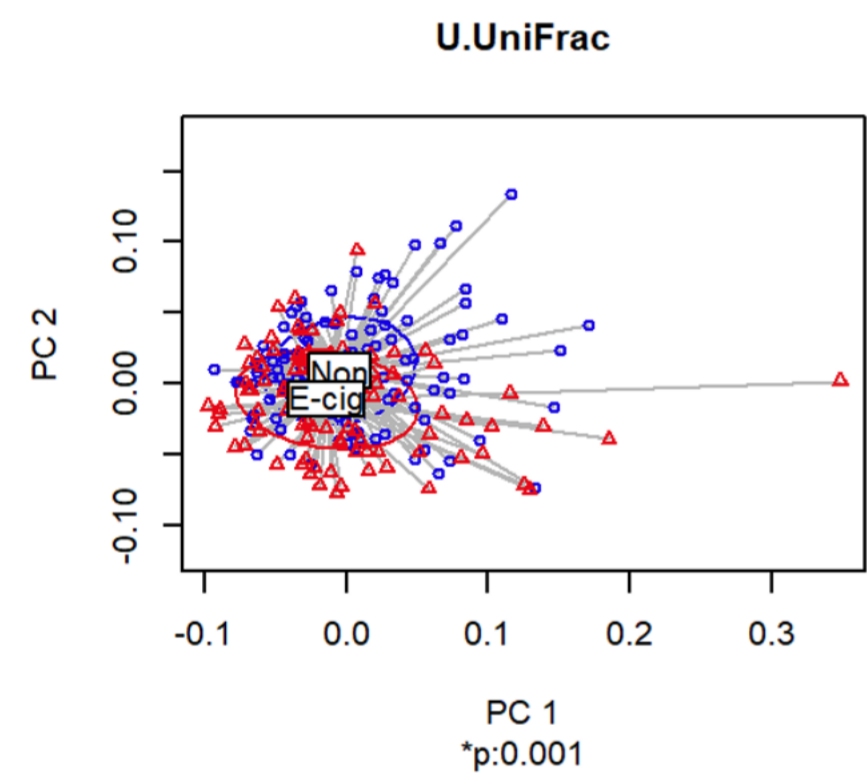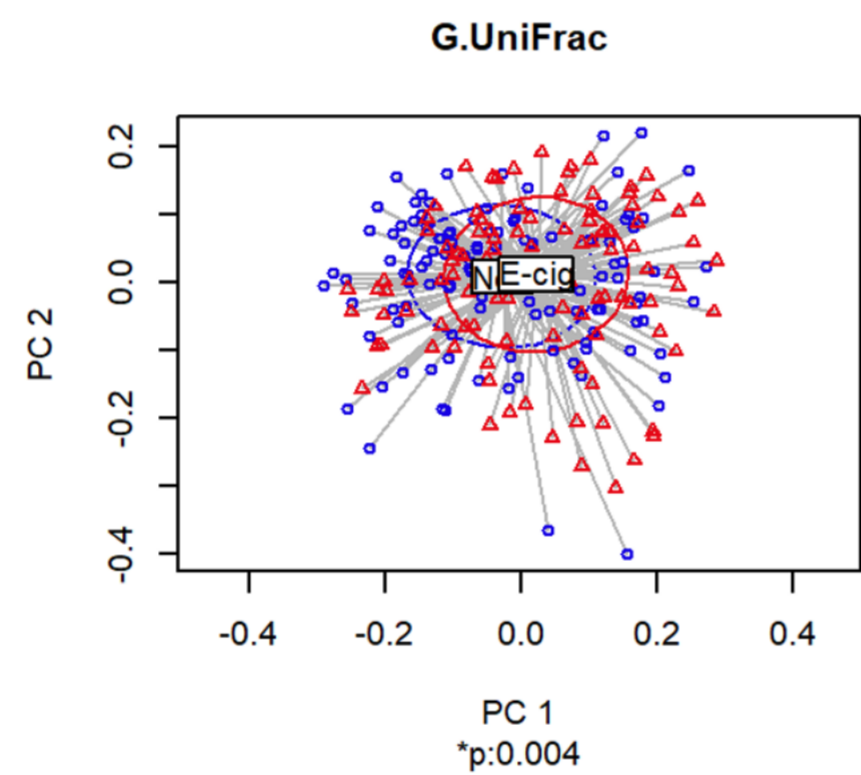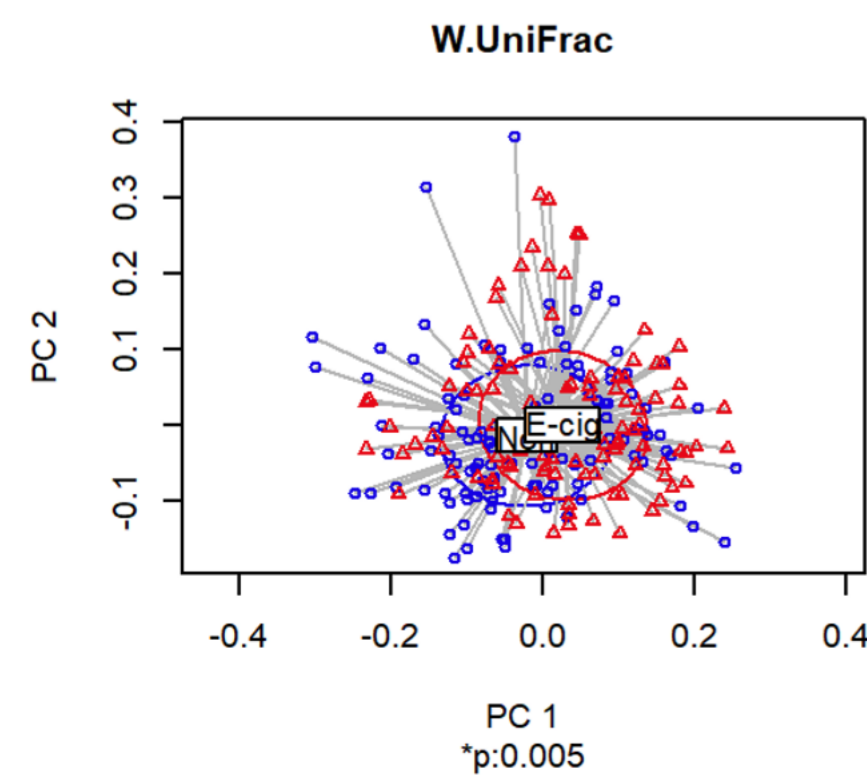

■ Non-smokers  
■ E-cigarette smokers

**B**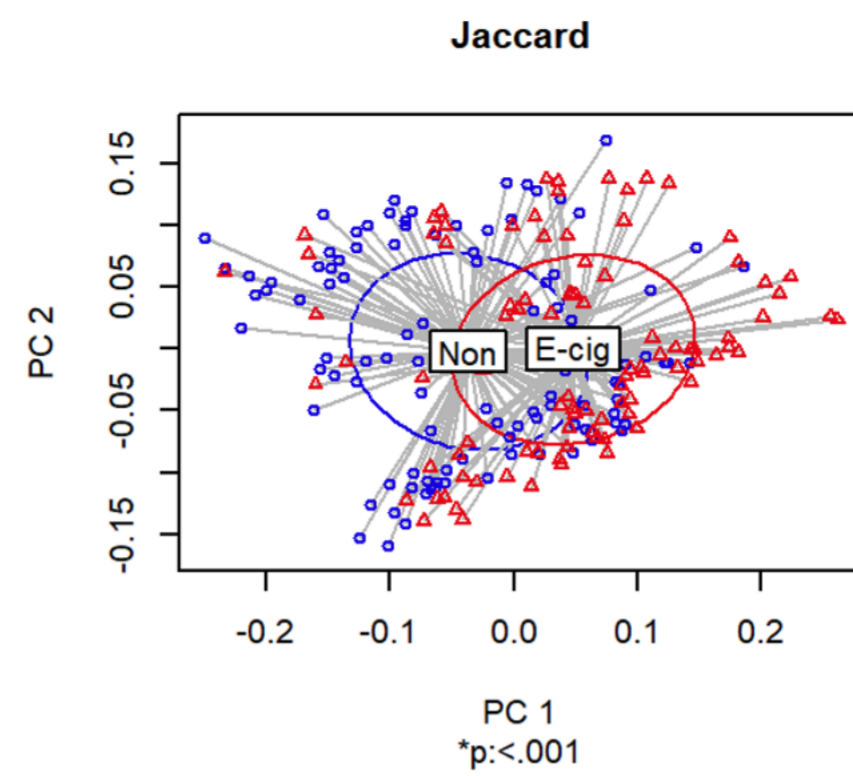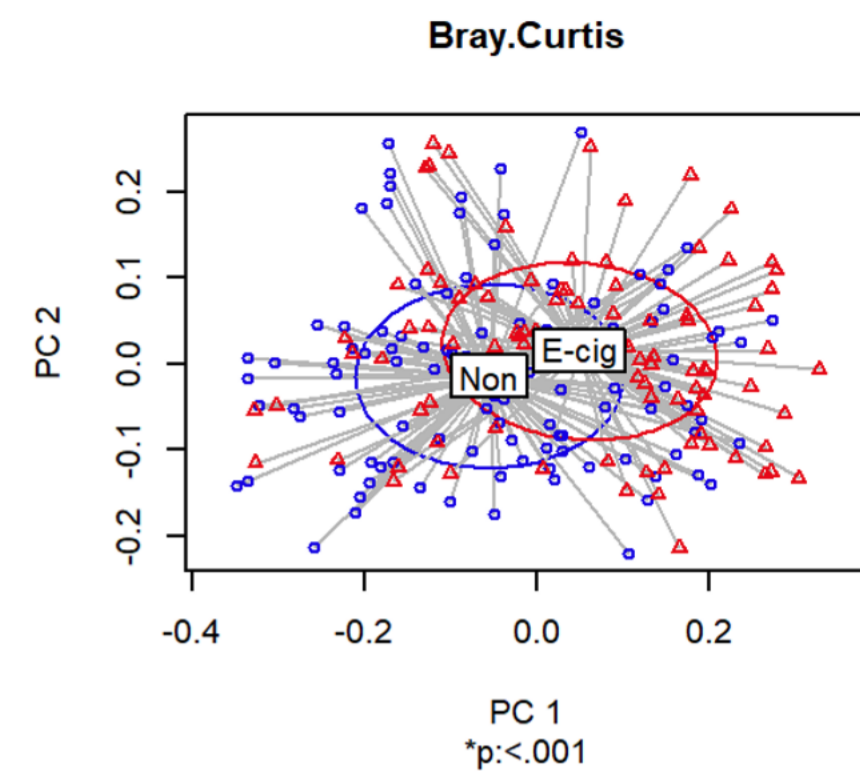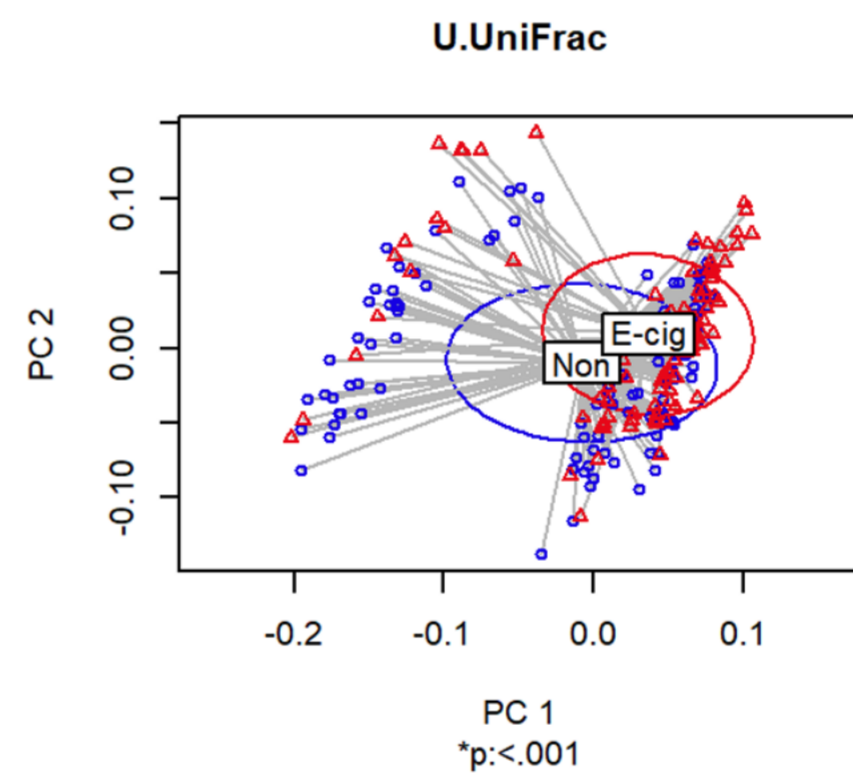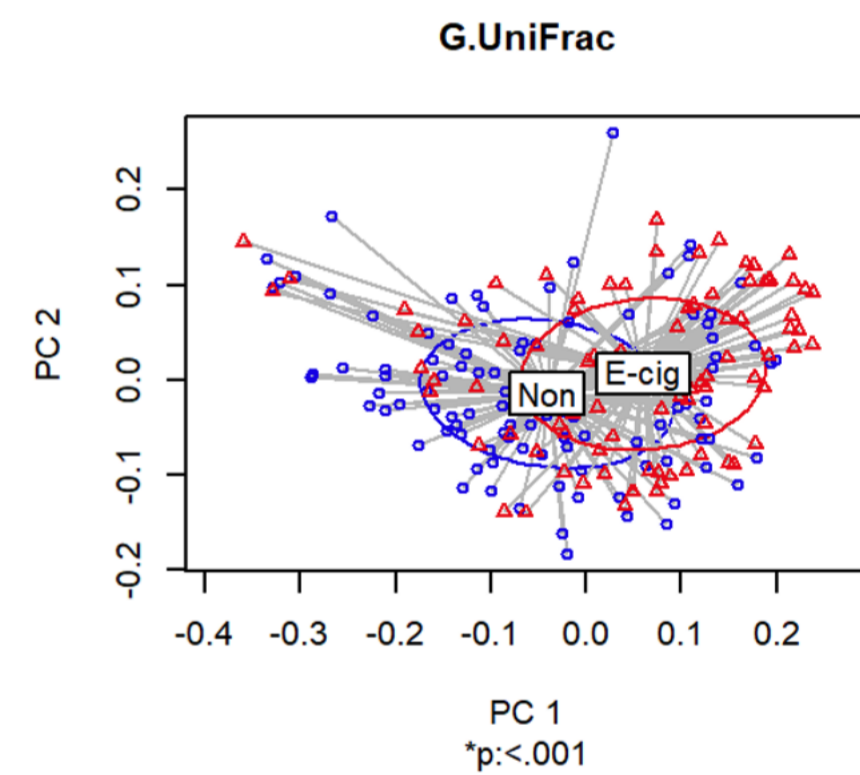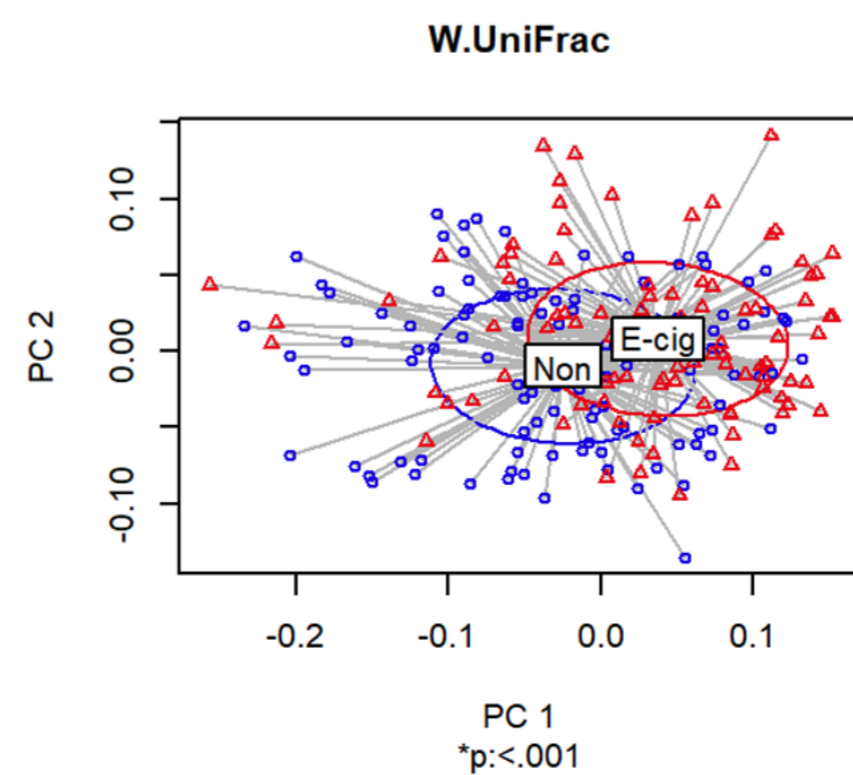

■ Non-smokers  
■ E-cigarette smokers

Supplement: Supplementary file 3 — Additional file 3: Figure S3. The results from unadjusted β-diversity analysis using saliva (A) and subgingival (B) samples. [file 12866_2023_2779_MOESM3_ESM.pdf]

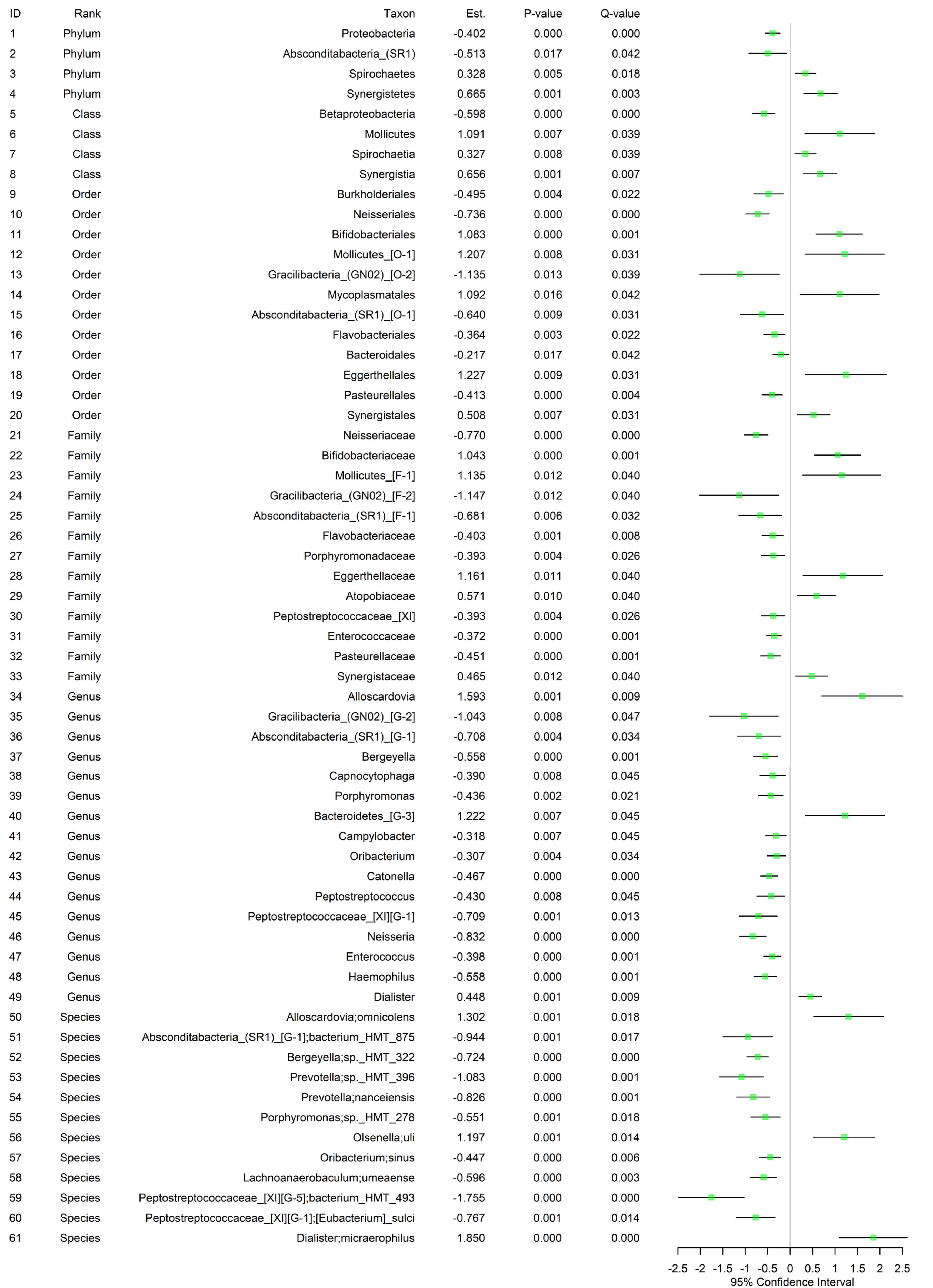

Supplement: Supplementary file 4 — Additional file 4: Figure S4. The results from unadjusted taxonomic differential abundance analysis using saliva samples. [file 12866_2023_2779_MOESM4_ESM.pdf]
