# Supplementary material for: The mediating roles of the oral microbiome in saliva and subgingival sites between e-cigarette smoking and gingival inflammation
Source: BMC Microbiol. 2023 Feb 2;23:35. doi: 10.1186/s12866-023-02779-z (PMC9893987; doi:10.1186/s12866-023-02779-z)

ID	Rank	Taxon	Est.	P-value	Q-value
1	Phylum	Actinobacteria	-0.467	0.004	0.012
2	Phylum	Synergistetes	0.746	0.002	0.006
3	Phylum	Proteobacteria	-0.561	0.000	0.001
4	Phylum	Spirochaetes	0.605	0.000	0.001
5	Class	Flavobacteriia	-0.577	0.000	0.001
6	Class	Bacteroidetes_[C-1]	0.760	0.014	0.043
7	Class	Actinobacteria	-0.528	0.001	0.004
8	Class	Synergistia	0.733	0.002	0.009
9	Class	Gammaproteobacteria	-0.617	0.001	0.004
10	Class	Betaproteobacteria	-0.722	0.000	0.001
11	Class	Spirochaetia	0.593	0.000	0.002
12	Class	Bacilli	-0.397	0.017	0.046
13	Order	Flavobacteriales	-0.544	0.000	0.000
14	Order	Bacteroidetes_[O-1]	0.788	0.015	0.045
15	Order	Actinomycetales	-0.492	0.000	0.002
16	Order	Corynebacteriales	-0.515	0.000	0.001
17	Order	Synergistales	0.769	0.004	0.014
18	Order	Pasteurellales	-0.542	0.003	0.014
19	Order	Burkholderiales	-1.198	0.000	0.000
20	Order	Neisseriales	-0.619	0.000	0.001
21	Order	Cardiobacteriales	-0.789	0.000	0.000
22	Order	Spirochaetales	0.629	0.001	0.003
23	Order	Lactobacillales	-0.394	0.007	0.022
24	Family	Flavobacteriaceae	-0.581	0.000	0.000
25	Family	Atopobiaceae	0.706	0.006	0.025
26	Family	Actinomycetaceae	-0.479	0.000	0.003
27	Family	Corynebacteriaceae	-0.553	0.000	0.001
28	Family	Micrococcaceae	-0.801	0.000	0.002
29	Family	Synergistaceae	0.733	0.005	0.024
30	Family	Pasteurellaceae	-0.577	0.002	0.009
31	Family	Burkholderiaceae	-1.045	0.000	0.000
32	Family	Neisseriaceae	-0.656	0.000	0.001
33	Family	Cardiobacteriaceae	-0.825	0.000	0.000
34	Family	Spirochaetaceae	0.593	0.001	0.005
35	Family	Enterococcaceae	-0.732	0.000	0.000
36	Family	Streptococcaceae	-0.398	0.007	0.028
37	Genus	Bergeyella	-1.023	0.000	0.000
38	Genus	Bacteroidetes_[G-3]	1.387	0.002	0.013
39	Genus	Porphyromonas	-0.377	0.003	0.017
40	Genus	Capnocytophaga	-0.683	0.000	0.000
41	Genus	Leptotrichia	-0.377	0.006	0.036
42	Genus	Actinomyces	-0.720	0.000	0.000
43	Genus	Corynebacterium	-0.711	0.000	0.000
44	Genus	Rothia	-0.959	0.000	0.001
45	Genus	Peptidiphaga	-0.847	0.001	0.006
46	Genus	Haemophilus	-1.021	0.000	0.001
47	Genus	Lachnospiraceae_[G-7]	1.899	0.001	0.008
48	Genus	Peptostreptococcaceae_[XI][G-1]	1.479	0.001	0.008
49	Genus	Kingella	-0.867	0.000	0.004
50	Genus	Neisseria	-0.675	0.003	0.017
51	Genus	Cardiobacterium	-0.987	0.000	0.000
52	Genus	Treponema	0.431	0.007	0.039
53	Genus	Enterococcus	-0.893	0.000	0.000
54	Genus	Streptococcus	-0.558	0.001	0.008
55	Species	Bergeyella;sp._HMT_322	-1.094	0.000	0.000
56	Species	Porphyromonas;sp._HMT_278	-0.524	0.003	0.039
57	Species	Alloprevotella;rava	0.799	0.002	0.029
58	Species	Prevotella;aurantiaca	-1.352	0.002	0.036
59	Species	Prevotella;melaninogenica	-0.594	0.005	0.041
60	Species	Alloprevotella;tannerae	0.763	0.005	0.041
61	Species	Leptotrichia;sp._HMT_212	-0.815	0.003	0.039
62	Species	Olsenella;uli	1.452	0.000	0.001
63	Species	Rothia;dentocariosa	-0.897	0.001	0.019
64	Species	Haemophilus;sputorum	-0.812	0.005	0.043
65	Species	Campylobacter;sp._HMT_044	1.294	0.003	0.039
66	Species	Lachnoanaerobaculum;umeaense	-0.786	0.000	0.006
67	Species	Stomatobaculum;sp._HMT_097	-0.680	0.000	0.009
68	Species	Peptostreptococcus;anaerobius	-0.824	0.000	0.003
69	Species	Peptostreptococcaceae_[XI][G-7];bacterium_HMT_081	1.127	0.005	0.041
70	Species	Peptostreptococcaceae_[XI][G-1];[Eubacterium]_infirmum	1.160	0.002	0.036
71	Species	Prevotella;oralis	-0.900	0.000	0.007
72	Species	Cardiobacterium;valvarum	-1.101	0.000	0.001
73	Species	Haemophilus;aegyptius	-1.083	0.006	0.043
74	Species	Treponema;sp._HMT_262	0.961	0.004	0.041
75	Species	Treponema;sp._HMT_927	1.045	0.003	0.039
76	Species	Treponema;sp._HMT_238	1.115	0.004	0.041
77	Species	Capnocytophaga;sp._HMT_863	-0.767	0.000	0.001
78	Species	Prevotella;nanceiensis	-0.850	0.000	0.007
79	Species	Dialister;pneumosintes	1.023	0.000	0.006
80	Species	Megasphaera;sp._HMT_123	1.161	0.004	0.041
81	Species	Streptococcus;anginosus	0.782	0.005	0.041
82	Species	Abiotrophia;defectiva	-0.707	0.006	0.047

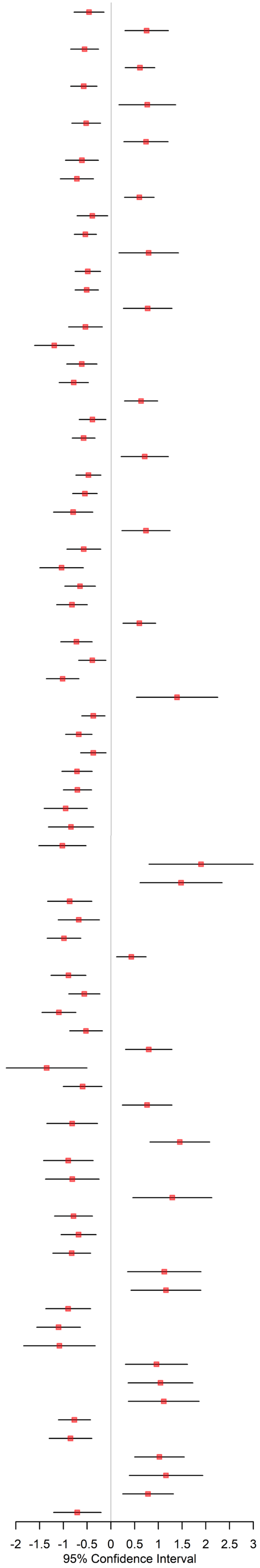

Supplement: Supplementary file 5 — Additional file 5: Figure S5. The results from unadjusted taxonomic differential abundance analysis using subgingival samples. [file 12866_2023_2779_MOESM5_ESM.pdf]
